# Impact of Education and Music Training on the Development of Abstract Thinking in the First Years of Schooling

**DOI:** 10.1162/OPMI.a.354

**Published:** 2026-05-29

**Authors:** Théo Morfoisse, Séverine Becuwe, Marie Palu, Cassandra Potier Watkins, Ghislaine Dehaene-Lambertz, Stanislas Dehaene

**Affiliations:** Collège de France, Paris, France; Cognitive Neuroimaging Unit, CEA, INSERM, Université Paris-Sud, Université Paris-Saclay, NeuroSpin Center, Gif/Yvette, France

**Keywords:** language of thought, cognitive development, music training, abstract reasoning, early education

## Abstract

Plato’s Republic, Einstein’s Theory of relativity, Vivaldi’s Four Seasons are all remarkable examples of humans’ unique ability to create and manipulate complex abstract structures, whether in language, mathematics, or music. Yet the mechanisms by which children develop such abstract thinking, and the role of education and structured experiences such as musical practice in shaping these abilities remains unclear. To explore these questions, we conducted cross-sectional behavioral experiments with 528 children aged 4 to 8, spanning four educational grades, half of whom participated in a violin training program, from age four. Two experiments examined how children encode, process, and compress auditory sequences and visual patterns, while a third examined their sensitivity to geometric regularities. Our results reveal the emergence of symbolic reasoning as early as the start of formal schooling, with deeper abstraction as a function of grade. By first grade, children encoded complex auditory sequences within a Language of Thought (LoT) similar to adults. Additionally, when confronted with quadrilaterals, children showed increasing sensitivity to geometric regularities, suggesting a developmental transition from perceptual to symbolic reasoning. However, we did not observe significant impact of musical practice on abstraction abilities across any of the domains tested. We discuss whether and how the impact of education and extracurricular activities such as music could be enhanced.

## INTRODUCTION

Whether in the domain of language, mathematics, or music, humans possess a remarkable ability to encode complex information abstractly by mentally restructuring it beyond its linear form (Chomsky, 1965; Dehaene et al., 2015; Hauser et al., 2002). For example, when reading a sentence or listening to a melody, we do not merely store a list or words or notes; instead, we reorganize them internally as nested, tree-like structures, using a language-like system (Boole, 1854; Chomsky, 1965; Dehaene et al., 2022; Goodman et al., 2008; Kemp & Tenenbaum, 2008; Spelke, 2022; Tenenbaum et al., 2011). The *Language of Thought* (LoT) framework has been proposed as one such potential system by which we do this (Dehaene et al., 2022; Fodor, 1975, 1983; Mandelbaum et al., 2022; Piantadosi & Jacobs, 2016; Piantadosi et al., 2012, 2016; Quilty-Dunn et al., 2023; Ullman et al., 2012). It posits that cognitive representations are constructed from a set of fundamental primitives that can be recursively recombined through key operations such as concatenation, repetition, and embedding.

Extensive behavioral and neuroimaging studies in adults support this framework (Al Roumi et al., 2021, 2023; Amalric et al., 2017; Dehaene et al., 2022; Ferrigno et al., 2020; Mills et al., 2023; Planton et al., 2021; Revencu et al., 2026; Sablé-Meyer et al., 2026; Wang et al., 2019). For instance, behavioral studies have shown that when participants are asked to memorize complex auditory sequences, their error rates were modulated by the complexity of the sequences as defined by a LoT-model (Al Roumi et al., 2023; Planton et al., 2021)—that is, by the minimum total number of primitive operations required to fully describe them (Chater & Vitányi, 2003; Feldman, 2000, 2003; Mathy & Feldman, 2012). This suggests that when the number of items exceeds working-memory capacities (e.g., 16-item sequences), adults compress these sequences into structured, language-like representations to support memorization. Beyond temporal sequences, humans also exhibit a propensity to encode mathematical and visual objects using abstract, recursive rules (Cheyette & Piantadosi, 2017; Dehaene et al., 2022; Feldman, 2000, 2003; Mathy & Feldman, 2012; Mills et al., 2023, 2024; Piantadosi et al., 2012; Pomiechowska et al., 2024; Sablé-Meyer et al., 2021, 2022, 2026). Recent work, for example, has demonstrated that a visual LoT model can properly explain human memory for complex geometric shapes such as a “square of triangles” (Sablé-Meyer et al., 2022), as well as the learning of many spatiotemporal patterns, from simple geometrical shapes to intricate mathematical graphs (Ciccione et al., 2026; Mills et al., 2023, 2024). More broadly, LoT models—and especially probabilistic ones—have also proven highly effective at explaining human learning as Bayesian program induction, in which learners search for the simplest compositional expression that accounts for the data (Goodman et al., 2008; Kemp & Tenenbaum, 2008; Piantadosi & Jacobs, 2016; Piantadosi et al., 2012; Tenenbaum et al., 2011).

While the *LoT* framework has shed light on adults’ ability to *represent* and *learn* information in a compressed and abstract way, the extent to which it can explain children’s behavior remains uncertain. Some studies suggest that infants and children exhibit early signs of compositional and logical abilities, which are key components of LoT (Cesana-Arlotti et al., 2018; Ciccione et al., 2026; Dautriche & Chemla, 2025; de Carvalho & Dautriche, 2025; Ekramnia et al., 2021; Feiman et al., 2022; Piantadosi & Aslin, 2016; Pomiechowska et al., 2024). Yet, other studies indicate that although these models capture aspects of children’s performance, their explanatory power falls short in comparison to that observed in adults (Mills et al., 2024; Sablé-Meyer et al., 2021), likely reflecting the fact that children are in a transitional phase, still refining their ‘mental algorithms’ (Siegler, 1996) and potentially using a mixture of simple perceptual strategies and emerging abstract representations. This raises the question of how the Language of Thought develops and to what extent it is shaped by education and experience (Cesana-Arlotti et al., 2018; Feiman et al., 2022; Piantadosi & Jacobs, 2016; Piantadosi et al., 2012, 2016).

To address these questions, we conducted a series of behavioral experiments with 528 children aged 4 to 8, spanning four educational grades. This range was deliberately selected to capture the developmental transition that occurs around the onset of formal schooling—a critical period during which children acquire foundational abilities in domains such as mathematics and reading. Across three experiments, we investigated how formal schooling shapes the development of symbolic abstraction. We utilized paradigms known to elicit Language of Thought (LoT) representations in adults, spanning auditory and visual sequence learning (Planton et al., 2021) as well as geometric reasoning (Sablé-Meyer et al., 2021).

To uncover the cognitive principles driving developmental changes in sequence and pattern encoding, we mapped longitudinal performance gains against a battery of computational models. We specifically tested the hypothesis that a Language of Thought (LoT) architecture, rooted in recursive compression, provides a superior fit for developmental data compared to simpler chunking or entropy-based metrics. We extended this comparative approach to geometric reasoning, contrasting a simple biological-inspired convolutional neural network (CNN) with a symbolic model defined by discrete geometric primitives such as parallelism and right angles. Previous research has shown that adults are highly sensitive to these regular properties, whereas baboons (and simple CNNs) do not single them out, treating a square and a trapezoid as similarly complex. By identifying which model best predicts children’s reasoning, we aim to reveal when and how symbolic, language-like representations begin to organize reasoning across both domains, potentially supporting an early and unified Language of Thought framework.

Beyond the impact of formal education, we also investigated whether learning another symbolic language, music, can enhance abstraction abilities. Since music can be conceptualized as a structured and abstract language, musical education and practice have often been proposed as potential sources of transfer to abstract reasoning, particularly in relation to mathematics (Bilhartz et al., 1999; Costa-Giomi, 2004; Holmes & Hallam, 2017; Holochwost et al., 2017; Mehr et al., 2013; Rickard et al., 2012; Schellenberg, 2004). Indeed, music, mathematics, and language all require the manipulation of abstract, symbolic, and recursive structures, and some of their underlying concepts may overlap. For instance, the hierarchy of note durations in music (whole, half, quarter, and eighth notes) echoes the mathematical concept of powers of two or fractions. Understanding an abstract concept in one domain (e.g., half-notes and quarter notes) may thus support comprehension of a corresponding concept in another (e.g., powers of two). An even stronger hypothesis would be that mastering abstract notions in one domain (such as music) might foster the ability to manipulate abstract structures in any domain (such as mathematics).

However, current empirical evidence for such cross-domain transfer remains limited (Bilhartz et al., 1999; Costa-Giomi, 2004; Holmes & Hallam, 2017; Holochwost et al., 2017; Mehr et al., 2013; Rickard et al., 2012; Schellenberg, 2004) and has mostly relied on academic or standardized measures. In contrast, the present work focuses on the more fundamental abstraction skills underlying the manipulation of structured, symbolic, and relational representations. These abilities, often viewed as core components of the LoT, are also considered foundational to the development of mathematical reasoning (Dautriche & Chemla, 2025; de Carvalho & Dautriche, 2025; Dehaene et al., 2022; Hochmann, 2022; Piantadosi et al., 2016, 2018; Pomiechowska et al., 2024). Because our tasks are less tied to formal schooling, they may provide a more sensitive probe of the cognitive mechanisms through which musical learning might influence abstract reasoning (see Banerjee et al., 2025 for similar considerations). We hypothesized that, if transfer exists, its effects would be strongest for auditory sequences (closest transfer) and progressively weaker for visual sequences and geometric abstraction (furthest transfer). These hypotheses are situated within a broader, highly debated and polarized body of research investigating whether musical training can improve a range of different non-musical domains, such as executive functions or language; while some researchers argue for significant cognitive benefits (Bigand & Tillmann, 2022; Jamey et al., 2024) others report only modest or null effects (Sala & Gobet, 2017, 2020; Schellenberg & Lima, 2024). We return to these conflicting findings in our discussion, interpreting our results through the lens of this broader literature on cross-domain transfer.

To empirically assess this hypothesis, we leveraged a preexisting ambitious musical education program called *Un Violon dans mon école* (A Violin in My School) and implemented in disadvantaged schools in the suburbs of Paris. In half of these schools, children receive weekly violin lessons for four consecutive years, from preschool through second grade, while the other half follow the standard curriculum, without formal musical instruction. School assignment was performed by the program, independently and prior to our study. Our sample included 272 children from the violin schools and 256 from the control schools, all of whom performed the three previously described tasks. In addition, because inhibitory control has been (often but not always) reported to be influenced by music training, we included a fourth task assessing this ability (Bolduc et al., 2021; Bugos & DeMarie, 2017; Frischen et al., 2019, 2021; Guo et al., 2018; Holochwost et al., 2017; Janus et al., 2016; Shen et al., 2019). We used a composite task, originally developed by Bunge et al. (2002), integrating a flanker task (congruent vs. incongruent) (Eriksen & Eriksen, 1974; Fan et al., 2002) and a go/no-go task.

## MATERIALS AND METHODS

### Participants

A total of 566 children were tested across two waves. In June 2023, 292 children were tested (115 from violin schools and 177 from control schools). In June 2024, 274 children were tested (190 from violin schools and 84 from control schools). Because 38 children participated in both sessions, and to avoid potential habituation bias, we excluded their second session data from the analyses. Table 1 reports the number of children tested by grade. All children attended suburban Paris schools that were part of the priority education network. Supplementary Material Table S1 details the number of children tested per school and each school’s social position index (SPI), reflecting the socioeconomic and cultural backgrounds of the families. Individual-level information (e.g., age, extracurricular activities) could not be collected. Throughout the article, children were sampled across grade levels. While we refer to differences between these groups as developmental, we acknowledge that such changes likely result from both neurobiological maturation and exposure to the formal school curriculum.

**Table 1.** Number of children in each grade. Ages denote the typical ranges associated with each French grade level sampled.

	Preschoolers	Kindergarteners	1st Graders	2nd Graders
	(4 to 5-y-olds)	(5 to 6-y-olds)	(6 to 7-y-olds)	(7 to 8-y-olds)
Violin	36	92	81	63
Control	54	66	101	35
Total	90	158	182	98

Ahead of the experiments, all parents received a form to return if they did not wish for their child to participate. Additionally, a consent form was collected from each child before testing began. While all authorized children were eligible to participate, only those with no known developmental disorders (e.g., dyslexia), based on the teacher’s reports, were included in the analyses.

#### Musical Program.

*Un violon dans mon école* is a music education program implemented in disadvantaged French schools in the suburbs of Paris (France). In half of these schools, who volunteered to participate in the program, children received mandatory weekly violin lessons from preschool to second grade (4 years). Children in the other half followed the standard curriculum without formal musical instruction, though these schools received budgetary rewards for educational resources like books. The schools were not randomized at the onset, but it was compulsory for all children to attend music lessons if they were in a violin school. The authors were not involved in the design or implementation of the *Un violon dans mon école program.*

Qualified teachers provided age-appropriate violin lessons, adapting the pedagogy over the four years. In the first year, children attended weekly 45-minute group sessions (12 students per group). In the following three years, they continued with the 45-minute sessions and additional two weekly 30-minute small-group sessions (4 to 6 children), for a total of 1 hour and 45 minutes of musical instruction per week. Year 1 introduces basic violin handling, posture, pizzicato, simple rhythms, melodies and foundational musical concepts. Year 2 emphasizes refining posture, exploring harmonics, using multiple fingers on the violin, and understanding nuances, tempo and phrasing. Year 3 deepens technical skills, including bowing techniques, articulation, playing on multiple strings, and reading music. Year 4 consolidates all skills, focusing on advanced bowing, finger mobility, playing complex melodies, and composing short pieces. Across all years, children develop autonomy in violin care, synchronization, and musical expression, with gradual exposure to classical repertoire.

Music lessons took place during school hours, replacing a portion of instructional time otherwise devoted to other subjects. Each teacher was free to organize their own schedule, so the domains that may been deprioritized varied across classrooms and schools. Nevertheless, as explained in more detail in the general discussion, a 2022–2023 survey of first-grade teachers (Pereira, 2025) suggests that while domains like visual arts were often displaced by the music intervention, core subjects—specifically French and mathematics—remained largely unaffected. This indicates that instruction in the domain most relevant to our experiments—mathematics—was preserved across groups.

### Procedure

All children were tested during regular school hours. Four experiments were conducted entirely on a tablet with headphones, lasting 30 to 40 minutes in total (5–7 minutes per task). The order of the experiments was randomized across participants and had no significant impact on performance. Ten experimenters administered the tests, each overseeing two to three children at a time. Task instructions were pre-recorded in video format to maximize autonomy, with experimenters ensuring proper tablet function and maintaining children’s focus.

## EXPERIMENT 1: AUDITORY SEQUENCE TASK

Experiment 1 evaluated the ability to detect patterns in auditory sequences and compress information in memory (Planton et al., 2021).

### Experimental Paradigm

**Figure 1.**
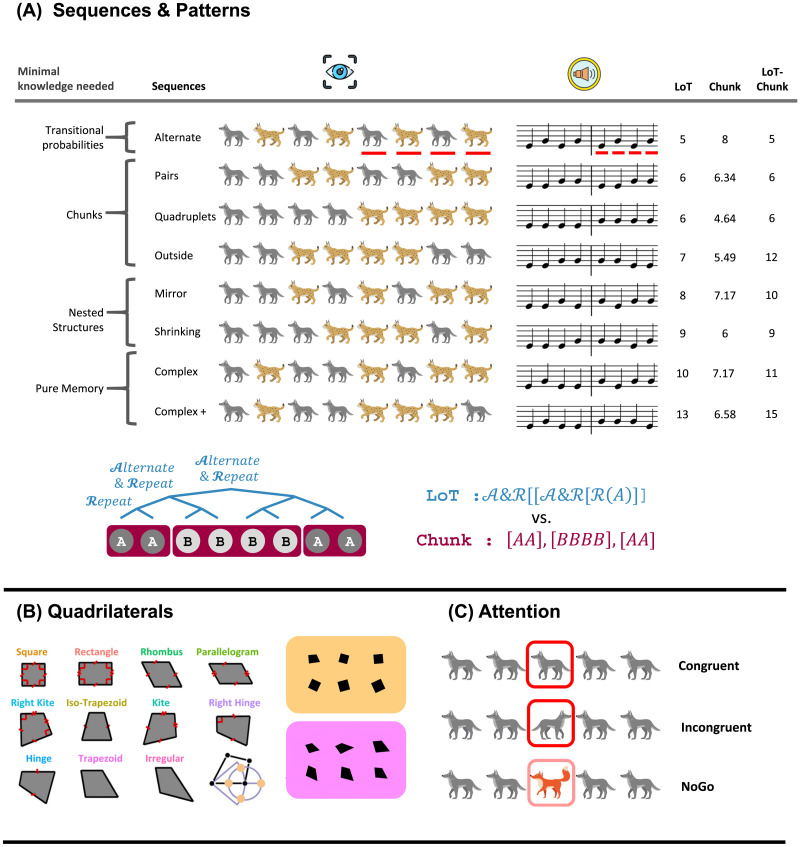
Experimental Paradigms. (A) Sequence Abstraction tasks: To assess symbolic encoding, children performed a same/different judgment task on pairs of eight-note auditory melodies or static visual patterns (8-animal row). In the columns on the right, sequences are categorized by their structural complexity based on three computational accounts: Language of Thought (LoT), Chunking, and LoT-Chunking. Complexity levels range from simple alternating patterns to “pure memory” sequences lacking recursive structure. Below, a representative LoT tree (blue) illustrates the recursive nesting strategy versus a flat chunking strategy (red) for the sequence [AABBBBAA]. (B) Quadrilateral geometry task: Using an odd-one-out paradigm, children identified a deviant shape among six quadrilaterals. The shapes varied systematically in geometric regularity, ranging from highly regular (e.g., square) to irregular forms. Deviants were created by a controlled shift of a single vertex (Sablé-Meyer et al., 2021). Two examples of trials are shown to the right of the 11 quadrilaterals: (top) square, (bottom) trapezoid. (C) Flanker Attention task: To measure selective elective and executive control, children indicated the facing direction of a central target animal. In congruent trials, distractors faced the same direction; in incongruent trials, distractors faced the opposite direction. No-Go trials (indicated by a distinct animal color) required response inhibition.

In this task, children heard two successive eight-note melodies and judged whether they were identical or whether the second melody deviated from the first by a single deviant note. The melodies were composed of only two pitches (one low and one high) and followed one of eight patterns, ranging from simple alternations (e.g., ABABABAB, where A and B represent the two notes) to complex sequences (Figure 1A). In trials where the melodies differed, one of the last four notes of the second melody was changed (A instead of B). Each melody lasted two seconds, separated by a one-second silence. Responses (same or different) were allowed from the onset of the second melody, with an additional two-second window afterward. A one-second pause preceded the next trial. The children were first familiarized with the task through eight sequences of increasing length, receiving auditory and visual feedback after each response. After training, the experiment included 32 trials: 16 sequences with identical melodies (8 sequences repeated twice), and 16 with different melodies were randomly presented. No feedback was provided.

### Data Analyses

We first conducted logistic regressions to determine whether error rates in each grade-level differed significantly from chance. Next, to explain performances, we evaluated several of the theoretical models proposed in the literature to describe sequence learning (see below) and compared their predictions, with a particular focus on whether the LoT models provided a better fit to children’s performance and whether it evolved with grades. For each grade and model, we performed a linear regression between the model’s predicted values and the average performance across participants for each sequence. When necessary, model fit was evaluated using the Akaike information criterion (AIC), which balances goodness of fit against model complexity (i.e., the number of predictors), with the lowest AIC value indicating the best-fitting model. Finally, we assessed the potential impact of the music program using two statistical approaches: a frequentist binomial mixed-model regression and a Bayesian logistic regression. Both models included the following predictors: violin training (violin vs. control), grade, SPI, and the complexity measures from the best-fitting model for the auditory task (LoT-Chunk) and the best-fitting model for the visual one (Chunk). Participants were included as a random effect in each model.

### Competing Models of Sequence Encoding

#### Language of Thought (LoT).

In the LoT framework, a sequence’s complexity reflects how simply it can be described using a small set of mental operations: concatenation, repetition, and recursive nesting (i.e., call of a subprogram). As in human language, a set of primitives (i.e., the basic elements or “atoms” of a given language) is defined upon which operations are applied. Here, only two primitives are needed: stay (+0) and change (+1), reflecting the two possible notes in each sequence. For example, the sequence AAAA could be described as a simple concatenation ([+0, +0, +0, +0]) or more compactly as a repetition ([+0]^4^). The shortest possible description (Minimal Description Length, or MDL) determines the sequence LoT complexity (Chater & Vitányi, 2003; Feldman, 2000, 2003; Mathy & Feldman, 2012).

#### Chunk Complexity.

This model assesses how well a sequence can be broken into repeated patterns or “chunks.” For example, the sequence *AAABAA* can be divided into 3 chunks—*AAA*, *B*, and *AA*. Mathematically, chunk complexity is expressed by the formula proposed in Mathy and Feldman (2012): $\sum_{i=1}^{K}$ *log*_2_(1 + *L*_*i*_), where *K* represents the total number of chunks and *L*_*i*_ denotes the length of the *i*th chunk. The chunk complexity of the sequence *AAABAA* is then: *log*_2_(4) + *log*_2_(2) + *log*_2_(3).

#### LoT-Chunk Complexity.

Combining the above two models, this metric calculates the simplest LoT-style description while preserving natural chunk boundaries. For example, the optimal LoT description of ABBAAB is [AB] [BA] [AB] (i.e., 3 repetitions of the stay-change program; LoT complexity = 5). Yet, this optimal description ignores the natural chunks BB and AA. The underlying intuition captured by this model is that humans group elements before compressing them (A [BB] [AA] B). This model has best explained memory performance in prior studies (Al Roumi et al., 2021; Planton et al., 2021).

#### Lempel-Ziv (LZ) Complexity.

Based on a data compression algorithm (Lempel & Ziv, 1976), this model counts how many unique patterns are found when scanning a sequence from left to right. For example, in the sequence *AAABAA*, the scan identifies three unique substrings: [A] [AA] [B], resulting in an LZ complexity of 3.

#### Sub-Symmetries.

This model counts the number of symmetrical patterns within a sequence (Alexander & Carey, 1968). For example, the sequence *AABBAB* has four symmetric sub-sequences: *AA*, *BB*, *BAB*, and *ABBA*.

#### Change Complexity.

This metric focuses on how much the sequence changes from one element to the next across its subsequences, capturing variation over time (Aksentijevic & Gibson, 2012).

**Shannon entropy** measures the uncertainty associated with a sequence by summing the uncertainties associated of all possible item pairs in the sequence (in our case AA, AB, BA, and BB). Mathematically, the entropy is formulated as:

$$E=-\left[P\left(A\right)\cdot P\left(A|A\right)\cdot \left({\text{log}}_{2}\left(P\left(A\right)\right)+{\text{log}}_{2}\left(P\left(A|A\right)\right)\right)+P\left(A\right)\cdot P\left(B|A\right)\cdot \left({\text{log}}_{2}\left(P\left(A\right)\right)+{\text{log}}_{2}\left(P\left(B|A\right)\right)\right)+P\left(B\right)\cdot P\left(A|B\right)\cdot \left({\text{log}}_{2}\left(P\left(B\right)\right)+{\text{log}}_{2}\left(P\left(A|B\right)\right)\right)+P\left(B\right)\cdot P\left(B|B\right)\cdot \left({\text{log}}_{2}\left(P\left(B\right)\right)+{\text{log}}_{2}\left(P\left(B|B\right)\right)\right)\right]$$

#### Algorithmic Complexity (Kolmogorov Complexity).

Kolmogorov complexity (KC) is an abstract and non-computable mathematical notion (roughly, the length of the shortest computer program capable of generating a given sequence on a universal Turing machine). Recently, Gauvrit, Delahaye, Zenil and Soler-Toscano proposed an approximation of KC based on the “coding theorem”, which relates a sequence’s algorithmic complexity to the probability that a universal machine would output it.

Table 2 presents the eight sequences along with the predictor values of their psychological complexity for each model.

**Table 2.** Complexity Scores assigned to each sequence according to the different models.

**Sequences**	**LoT**	**Chunk**	**LoTChunk**	**LZ**	**Subsymmetries**	**Change**	**Entropy**	**Algorithmic**
ABABABAB	5	8	5	4	12	2.59	1	19.84
AABBAABB	6	6.34	6	5	8	2.51	1.96	22.62
AAAABBBB	6	4.64	6	5	12	1.88	1.41	21.9
AABBBBAA	7	5.49	12	5	10	2.99	1.86	22.47
AABABABB	8	7.17	10	4	8	3.61	1.86	22.07
AAABBBAB	9	6	9	5	8	4.11	1.96	22.17
ABAABABB	10	7.17	11	5	7	3.7	1.86	21.58
ABAABBBA	13	6.58	15	5	7	4.25	1.96	21.82

### Results

#### Error Rates.

Children’s performance improved progressively with grade level, with average error rates of 48 ± 7% for preschoolers (PS), 44 ± 6% for kindergarteners (KG), 40 ± 6% for first graders (1stG), and 35 ± 7% for second graders (2ndG). Across all grades, based on logistic regressions, children’s performance exceeded chance level: PS (*z* = 2.68, *p* = .007), KG (*z* = 7.52, *p* < .001), 1stG (*z* = 13.8, *p* < .001), 2ndG (*z* = 15.3, *p* < .001).

**Figure 2.**
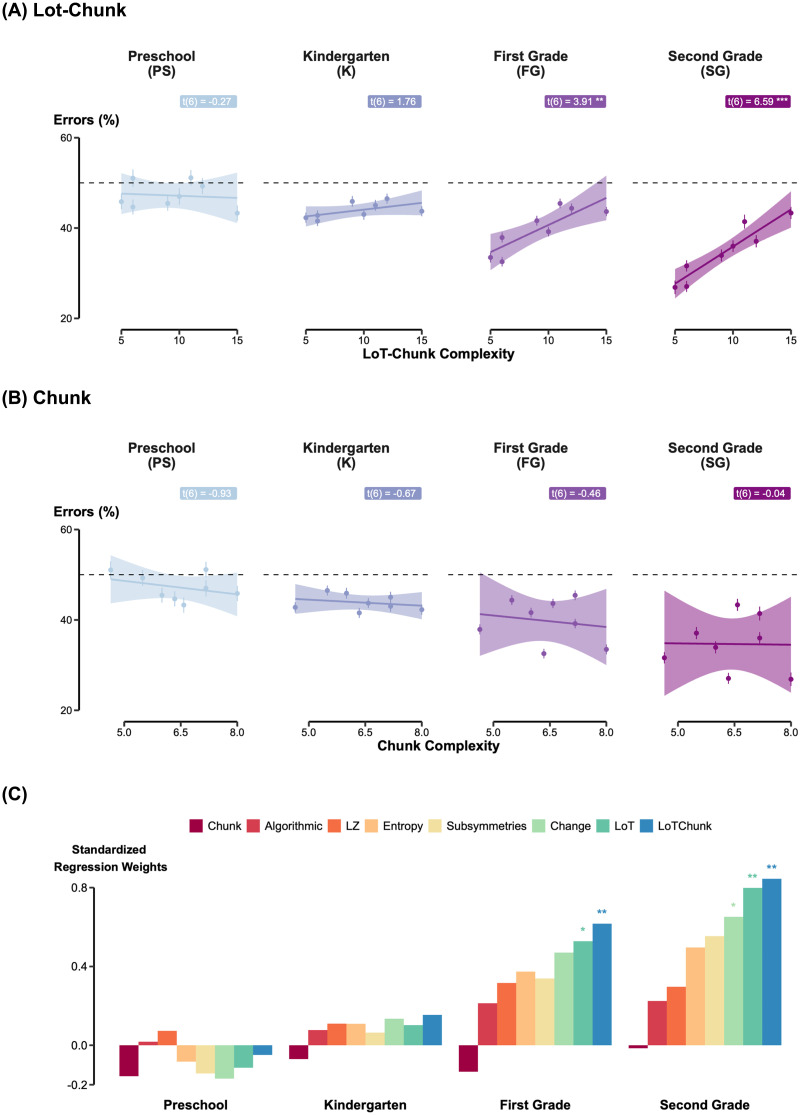
LoT-Chunk Complexity is the best predictor of error rates with auditory sequences. (A–B) Error Rates as a Function of Complexity. Mean error percentages across the eight sequence types are plotted for each grade level against LoT-Chunk complexity (A) and Chunk complexity (B). While performance at preschool and kindergarten levels remains near chance (50%), a robust linear relationship with LoT-Chunk complexity emerges in 1st and 2nd grade, whereas chunk complexity fails to capture the developmental trend. Shaded areas represent 95% confidence intervals. (C) Model Comparison Across Grades. Standardized regression weights for eight computational models (Chunk, Algorithmic, LZ, Entropy, Sub-symmetries, Change, LoT, and LoT-Chunk) show their relative predictive power for children’s performance. As children progress through grades, purely statistical models (e.g., Entropy, LZ) are increasingly outperformed by structured, Language of Thought-based frameworks. Asterisks indicate significant predictive weights (*p < .05, **p < .01).

To understand grade-related changes beyond basic error rates, we evaluated the different models described above (Table 2). A significant linear relationship between LoT complexity and error rates emerged from first grade onward (PS: (*t*(6) = −0.65, *p* = .54; KG: *t*(6) = 1.02, *p* = .35; 1stG: *t*(6) = 2.58, *p* = .042; 2ndG: *t*(6) = 4.69, *p* = .003) (Supplementary Material Figure S1A). An even stronger effect was observed for LoT-Chunk complexity (PS: *t*(6) = –0.27, *p* = .79; KG: *t*(6) = 1.76, *p* = .13; 1stG: *t*(6) = 3.91, *p* = .008; 2ndG: *t*(6) = 6.59, *p* = .001), accompanied by consistently better model fits (AIC = 16.58 vs. 12.40 for 1stG; 13.64 vs. 9.11 for 2ndG) (Figure 2A). These results indicate that higher LoT or LoT-Chunk complexity made the similarity-judgment task increasingly difficult. Both LoT-based measures were the strongest predictors of first and second graders’ performance, outperforming alternatives such as chunking models, entropy, and algorithmic complexity (Figures 2B and 2C). For 2nd graders for example, the best predictor was the LoT-Chunk (AIC = 9.10), followed by LoT (AIC = 13.65), Change (AIC = 20.04), Sub-symmetries (AIC = 22.17), Entropy (AIC = 23.08), LZ (AIC = 25.05), Algorithmic (AIC = 25.46) and finally Chunk (AIC = 25.97). In contrast, none of the complexity models, including probabilistic models, accounted for significant variance in the performance of younger children (preschoolers and kindergarteners).

#### Response Times.

RTs increased with grade level, averaging 2,198 ms for PS, 2,399 ms for KG, 2,617 ms for 1stG, and 2,794 ms for 2ndG (only correct trials included; Supplementary Material Figure S1B). RTs were largely uncorrelated with LoT-Chunk complexity (PS: *t*(6) = 0.36, *p* = .73; KG: *t*(6) = 2.20, *p* = .07; 1stG: *t*(6) = –0.78, *p* = .47; 2ndG: *t*(6) = 0.84, *p* = .43).

#### Music Training.

Lastly, we performed both a frequentist binomial mixed-effect regression and a Bayesian logistic regression. In both models, group (violins vs. controls), grade, SPI, LoT-Chunk and Chunk complexities were included as predictors, with participant modeled as a random-effect (Table 3, Supplementary Material Figure S1C–D). Overall, violin students performed slightly better than controls (*p* = .061), showing lower average error rates (40.5% vs. 42.1%), although the Bayesian analysis provided only anecdotal support for this difference (BF_10_ = .60 < 1, i.e., favoring the null hypothesis). This group difference did not increase with grade level (*p* = .11, BF_10_ = .49). Crucially, the performance of violin children and controls varied similarly as a function of sequence LoT-complexity (*p* = .92, BF_10_ = .037), and this similarity remained stable across grade levels (*p* = .53, BF_10_ = .046) (Supplementary Material Figure S1C).

**Table 3.** Parameter estimates from a frequentist binomial mixed-effects regression and a Bayesian logistic regression: *Errors* ∼ *Group* * *Grade* * (*LoTChunk* + *Chunk* + *SPI*) + (1|*Subject*). The predictors Grade, SPI, Lot-Chunk and Chunk were standardized prior to the regression. BF_10_ = Bayes Factor.

**Predictor**	**Estimate**	**Std. Error**	***Z*-value**	***P*-value**	**BF_10_**
(Intercept)	−0.428	0.042	−10.289	<.001 (***)	6.67e7
Group	0.228	0.122	1.871	.061	.60
**Grade**	**−0.212**	**0.036**	**−5.885**	<.001 (***)	**2.97e3**
**Lot-Chunk**	**0.120**	**0.025**	**4.722**	<.001 (***)	**478.07**
Chunk	−0.011	0.026	−0.421	.674	.028
SPI	−0.031	0.037	−0.839	.402	.050
Group × Grade	0.258	0.163	1.586	.113	.49
Group × LoT-Chunk	0.004	0.036	0.103	.918	.037
Group × Chunk	−0.021	0.037	−0.567	.571	.042
Group × SPI	−0.153	0.142	−1.078	.281	.23
**Grade × LoT-Chunk**	**0.071**	**0.026**	**2.759**	.006 (**)	**1.06**
Grade × Chunk	0.015	0.026	0.569	.569	.030
Grade × SPI	−0.037	0.047	−0.775	.438	.064
Group × Grade × LoT-Chunk	0.023	0.036	0.628	.530	.046
Group × Grade × Chunk	−0.033	0.037	−0.890	.373	.055
Group × Grade × SPI	−0.168	0.192	−0.879	.380	.26

### Discussion

Planton et al. (2021) found that adults’ performance in memorizing complex auditory sequences was strongly modulated by the LoT-complexity of the sequence. In other words, when the number of items exceeded their working memory capacities, adults compressed the sequences into structured, language-like representations (e.g., *ABABABAB* as four repetitions of alternating A and B) (Al Roumi et al., 2021; Planton et al., 2021).

The present results replicate and extend these conclusions to children, revealing that performance was jointly shaped by sequence complexity and grade level, whereas musical training exerts only a minimal effect. Children’s performance improved steadily across grades and from first grade onward their accuracy correlated strongly with the two complexity measures related to the LoT hypothesis (i.e., LoT and LoT-Chunk complexities). Among all tested frameworks, these models best capture first- and second- graders’ performance, outperforming alternative models based on chunking, entropy, sub-symmetries or even more sophisticated models such as algorithmic complexity. This pattern suggests that by early schooling, children begin to rely on genuinely structural representations. They do not appear to memorize sequences simply by partitioning them into chunks, or by tracking the number of transitions, their probabilities, the number of symmetries or changes. Instead, their performance indicates a growing ability to form recursive, nested, and compressed representations of sequences—hallmarks of a symbolic and hierarchical mode of cognition. Such results resonate with Chomsky’s generative framework and Fitch’s “dendrophilia” hypothesis, which propose that humans naturally infer hierarchical tree structures from linear input even in simple contexts (Chomsky, 1957; Fitch, 2014). While LoT-related complexities provided the best fit for first and second graders’ performances, the LoT-Chunk complexity appeared to be a better estimator than LoT alone, consistent with adult results (Al Roumi et al., 2023; Planton et al., 2021). This indicates that the optimal internal description of a sequence preserves its chunks structure: humans may initially segment the sequences into chunks before compressing them through symbolic operations.

We also explored whether cognitive strategies may shift with age, such that different models would capture performance at different grade level. However, none of the models reached significance in preschoolers and kindergarteners. Since these younger children struggled with the task and performed around chance level for all sequences, it remains difficult to draw any definitive conclusions. It is plausible that with shorter sequences or longer exposure times, a relationship between sequence complexity and performance might have emerged. Future studies using implicit, easier, or passive paradigms could help clarify the developmental trajectory of abstract reasoning abilities prior to schooling. Evidence from previous research already points in this direction. Preschool-aged children can display rudimentary forms of abstraction (Pomiechowska et al., 2024). For example, Mills et al. (2024) reported that 4–6-year-olds can learn to anticipate a wide range of visuospatial sequences, including linear functions and geometrical patterns, with their behavior best predicted by a LoT model—albeit with lower explanatory power than in adults.

Beyond the developmental perspective, we also examined the influence of musical practice on such abstraction abilities. Children learning the violin slightly outperformed control ones overall, particularly in first and second grade, suggesting a marginal advantage in memorizing auditory sequences. However, this difference remained at the level of a trend, and Bayesian analyses indicated that any effect was negligible. Furthermore, musical practice did not appear to enhance abstract reasoning: both groups showed similar performance patterns as sequence complexity increased, suggesting that violin children and controls relied on comparable LoT-based strategies. Thus, while musical training may refine auditory processing, we found no evidence that it strengthens sequence-abstraction abilities, consistent with the general findings of the existing literature (Bilhartz et al., 1999; Costa-Giomi, 2004; Holmes & Hallam, 2017; Holochwost et al., 2017; Mehr et al., 2013; Rickard et al., 2012; Schellenberg, 2004).

## EXPERIMENT 2: VISUAL PATTERN TASK

The second experiment, which tested young children’s ability to encode abstract regularities in visual patterns, further explored the possibility of transfer from music training to abstract reasoning. It also allowed us to investigate whether children employ the same symbolic strategies consistent with a Language of Thought, to encode static visual structures as they do for temporal auditory sequences.

### Experimental Paradigm

The design of Experiment 2 followed that of Experiment 1 but was adapted for the visual modality (Figure 1A). A key methodological departure from the first experiment was the simultaneous presentation of all eight items, in contrast to the sequential delivery used previously.

Children viewed two successive fixed animal patterns (8-animal rows) and judged whether the two patterns were identical. Each pattern was composed of two different animals arranged according to one of eight possible structures, ranging from simple alternations (e.g., ABABABAB, where A and B denote two different animals) to more complex patterns (Figure 1A). In non-identical trials, one of the last four animals in the second pattern was replaced by an unexpected one (e.g., A instead of B). Each pattern was presented with all animals simultaneously for two seconds, separated by a 1-second gray screen. Children could respond either during the presentation of the second pattern or within an additional 2-second gray screen response window. A final 1-second gray screen preceded the next trial. Before the main task, children completed a familiarization phase with eight practice patterns of increasing length, during which auditory and visual feedback indicated response accuracy. The test phase included 32 trials: 16 identical trials (8 patterns, each presented twice), and 16 non-identical trials. No feedback was provided during this phase.

### Data Analyses

The analyses followed the same procedures as those used in Experiment 1.

### Results

#### Error Rates.

As in Experiment 1, there was a clear grade-related progression, with average error percentages decreasing from 52 ± 7% for PS, 50 ± 6% for KG, 43 ± 8% for 1stG, and 31 ± 8% for 2ndG. Performance was significantly above chance from first grade onward (PS: *z* = −0.13, *p* = .89; KG: *z* = 1.64, *p* = .10; 1stG: *z* = 9.92, *p* < .001; 2ndG: *z* = 20.1, *p* < .001).

**Figure 3.**
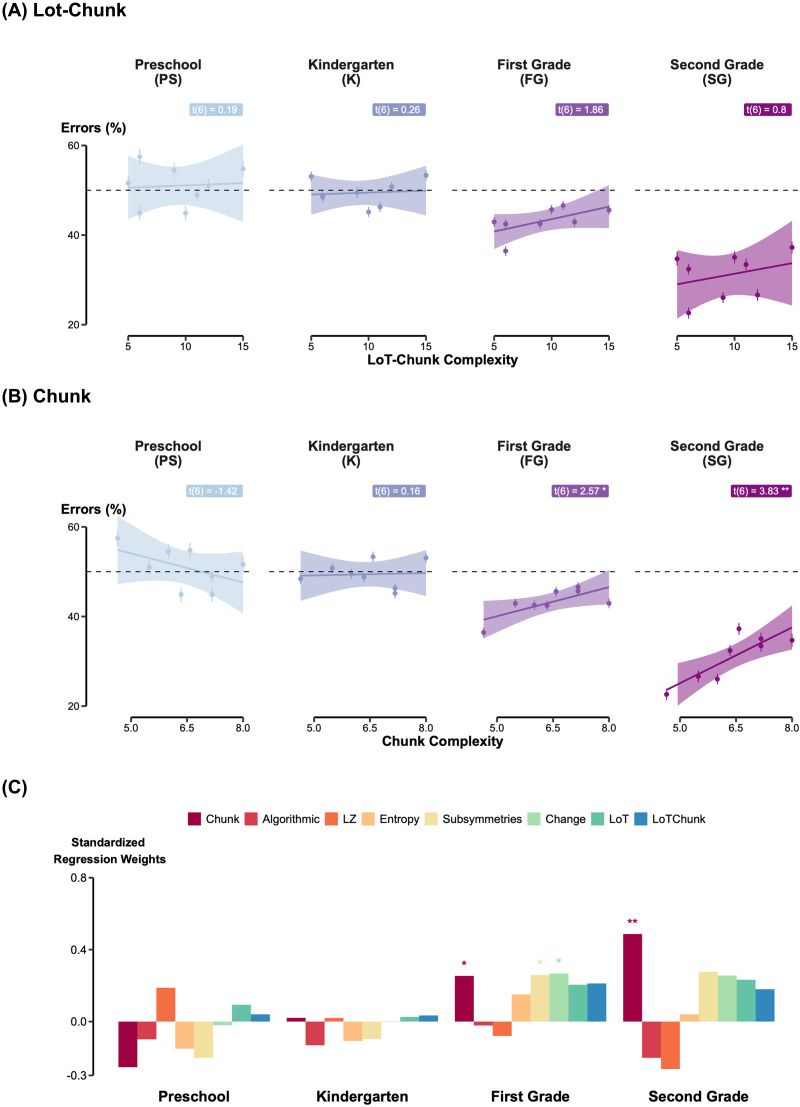
Chunk Complexity, rather than Lot-Chunk, predicts error rates in visual patterns. (A–B) Error Rates as a Function of Complexity. Mean error percentages for the eight visual patterns are plotted by grade against LoT-Chunk complexity (A) and Chunk complexity (B). Shaded areas represent 95% confidence intervals. (C) Model Comparison Across Grades. Standardized Regression weights from eight linear regressions performed between children’s average performance and model predictions, within each grade. Unlike auditory encoding, where LoT-based models gain dominance in later grades, visual sequence judgment in children appears to rely on chunk-based grouping strategies.

However, unlike in the auditory modality, *LoT*-related complexities did not adequately account for children’s performance at any grade (Figure 3A and Supplementary Material Figure S2A). For example, correlations with LoT-Chunk complexity were negligible even in the higher grades (all *p* > .10). Instead, models quantifying pattern features, such as the number or transitions, the probability of transitions, the number of symmetries or of changes, provided better explanations. Among these alternatives, the *Chunk model*, which quantifies the extent to which a sequence can be partitioned into subsequences of similar elements, emerged as the most explanatory model for first and second graders (PS: *t*(6) = –1.42, *p* = .21; KG: *t*(6) = 0.15, *p* = .88; 1stG: *t*(6) = 2.57, *p* = .042; 2ndG: *t*(6) = 3.83, *p* = .009; Figure 3B). For 2nd graders for example, the best predictor was the Chunk model (AIC = 8.96), followed by sub-symmetries (AIC = 16.79), LZ (AIC = 17.00), Change (AIC = 17.12), LoT (AIC = 17.47), LoT-Chunk (AIC = 18.05), Algorithmic (AIC = 18.83) and finally Entropy (AIC = 18.83). As in Experiment 1, none of the models significantly explained performance in younger children (PS and KG). Figure 3C summarizes the standardized regression estimates for all theoretical models tested.

#### Response Times.

RTs tended to increase with grade, averaging 1,550 ms for PS, 1,484 ms for KG, 1,609 ms for 1stG, and 1,668 ms for 2ndG (only correct trials). RTs were also uncorrelated with LoT-Chunk complexity PS: *t*(6) = –0.90, *p* = .40; KG: *t*(6) = 1.95, *p* = .10; 1stG: *t*(6) = –0.87, *p* = .42; 2ndG: *t*(6) = –0.92, *p* = .39 (Supplementary Material Figure S2B).

#### Music Training.

As in Experiment 1, we performed both a frequentist binomial mixed-model regression and a Bayesian logistic regression. In both models, group (violins vs. controls), grade, SPI, Lot-Chunk and Chunk complexities were included as predictor variables, with participant as a random effect (Table 4). No significant overall differences emerged between violin children and controls (Group: *p* = .72, BF_10_ = .13, error rates 41.5% for violins vs. 43.5% for controls; Group × Grade: *p* = .71, BF_10_ = .17). The performance of both groups varied similarly as a function of sequence Chunk complexity (*p* = 22, BF_10_ = .076), and this similarity remained stable across grades (*p* = .27, BF_10_ = .061; Supplementary Material Figure S2C). The same conclusions applied to the interactions involving LoT-Chunk complexity (Supplementary Material Figure S2D).

**Table 4.** Parameter estimates obtained from a frequentist binomial mixed-effects regression and a Bayesian logistic regression: *Errors* ∼ *Group* * *Grade* * (*LoTChunk* + *Chunk* + *SPI*) + (1|*Subject*). The predictors Grade, Lot-Chunk, Chunk and SPI were standardized prior to the regression. BF_10_ = Bayes Factor.

**Predictor**	**Estimate**	**Std. Error**	***Z*-value**	***P*-value**	**BF_10_**
(Intercept)	−0.335	0.049	−6.847	<.001	3.62e3
Group	−0.049	0.136	−0.359	.719	.13
**Grade**	**−0.359**	**0.042**	**−8.451**	<.001 (***)	**1.23e6**
**LoT-Chunk**	**0.062**	**0.025**	**2.485**	.013 (*)	**.56**
**Chunk**	**0.089**	**0.025**	**3.530**	<.001 (***)	**8.68**
**SPI**	**−0.115**	**0.044**	**−2.626**	.009 (**)	**1.29**
Group × Grade	−0.065	0.174	−0.371	.711	.17
Group × LoT-Chunk	−0.012	0.036	−0.349	.727	.037
Group × Chunk	−0.044	0.036	−1.220	.223	.076
Group × SPI	0.198	0.156	1.271	.204	.34
Grade × LoT-Chunk	0.029	0.026	1.121	.262	.046
**Grade × Chunk**	**0.099**	**0.026**	**3.798**	<.001 (***)	**35.80**
Grade × SPI	0.048	0.057	0.849	.396	.087
Group × Grade × LoT-Chunk	0.005	0.036	0.149	.882	.035
Group × Grade × Chunk	−0.040	0.037	−1.103	.270	.061
Group × Grade × SPI	0.194	0.204	0.951	.342	.30

### Discussion

While Experiment 1 showed that children’s auditory performance followed the predictions of the LoT model, Experiment 2 painted a different picture. Although visual performance improved with age and exceeded chance from first grade onward, essentially mirroring the auditory findings, it was better explained by chunking than by LoT complexity. Why, then, should two tasks built on the same pattern hierarchy yield such distinct outcomes?

This discrepancy is unlikely to result from task engagement, as performance levels were comparable across both modalities. It may instead stem from the modality itself: auditory processing is generally superior to vision for encoding temporal information (Guttman et al., 2005). However, modality alone may be an insufficient explanation, because Planton et al. (2021) demonstrated that adult’s performance with sequences of eight items, whether visual or auditory, was aligned with LoT complexity, with strong correlations across the two modalities. These findings point to a shared amodal mechanism in adults, that may not yet be fully functional or utilized in children. A more plausible explanation concerns the temporal structure of the task. Unlike the auditory task, where stimuli unfolded sequentially over time, the visual information was presented as a static pattern. While unpublished data from our lab suggest that adults remain sensitive to LoT measures even with static inputs, children may adopt a more restricted strategy. Specifically, they may focus on the last four items of the sequence—where the change occurs—and base their similarity judgments mainly on this final segment rather than on the full structure. In this scenario, the information load would not exceed working memory capacity, making a simple chunking strategy sufficient to perform the task. Unfortunately, the lack of variability in four-item sub-sequences prevents a formal model comparison between chunking and LoT models. We therefore tentatively conclude that, from preschool to second grade, children may rely on simpler models of visual patterns, with the Chunk model providing the best account of their behavior. While this interpretation is consistent with a body of prior research showing that chunking is widely utilized in memory strategies (Chekaf et al., 2016; Cowan, 2001; Mathy & Feldman, 2012; Miller, 1956), additional studies comparing simultaneously versus sequentially presented visual stimuli will be needed to draw definitive conclusions.

Beyond the developmental perspective, we also explored the potential influence of musical practice on these abilities. While music was a priori more likely to have an impact on auditory abstraction abilities, we hypothesized that it might also improve visual abstraction, through cross-modal or even amodal mechanisms. However, we found no differences between groups, suggesting that musical training does not enhance visual working memory. Additionally, the impact of pattern complexity did not differ between groups, indicating that musical practice has no discernible effect on either abstraction or chunking abilities. This result supports the interpretation that the modest advantage observed in the auditory modality likely reflected improved auditory processing rather than a broader enhancement of sequence abstraction.

## EXPERIMENT 3: QUADRILATERALS TASK

Experiment 3 evaluated the development of young children’s ability to perceive geometric regularities in quadrilateral shapes and also examined whether musical training—often assumed to strengthen symbolic or abstract processing—might influence their performance in this domain.

### Experimental Paradigm

This task followed a previous published odd-one-out paradigm (Sablé-Meyer et al., 2021) in which participants had to quickly identify a deviant shape among a set of six. All shapes were quadrilaterals, ranging from highly regular (a square) to fully irregular (an arbitrary quadrilateral lacking any geometrical properties) (Figure 1B). For each reference quadrilateral, four deviant versions were generated by shifting the bottom-right vertex by a fixed distance, either along the bottom edge or along a circle centered on the bottom-left vertex. On each trial, one of the 11 possible reference quadrilaterals was selected. Five instances of it, varying in scale and orientation (e.g., five rectangles), were presented simultaneously alongside a single deviant (e.g., a rectangle with a displaced bottom-right vertex) in a 3 × 2 grid. The position of the deviant was randomized, and six levels of rotation and scale were pseudo-randomly assigned across the six shapes. Participants had to click on the deviant shape as fast and accurately as possible. Before the main task, children completed a familiarization phase with five practice trials in which they had to find the odd animal among five others. The test phase included a total of 44 trials (4 deviants × 11 quadrilaterals).

### Competing Models

In Sablé-Meyer et al. (2021), the authors contrasted two classes of models of geometric shape perception: a convolutional neural network (CORnet) modeling the ventral visual pathway, treating geometrical shapes as holistic visual objects; and a symbolic model considering geometrical shapes as a list of discrete geometric primitives (e.g., right angles, parallelism, symmetry). This model is called *symbolic* because it considers quadrilaterals as mental structures built out compositionally of a very small set of initial primitives (e.g., parallelism; Dehaene et al., 2022). For the CNN model, the complexity of each quadrilateral and its deviants was quantified using the L2-norm between their activation vectors in the network’s final layer. In the symbolic model, shapes are represented by vectors of geometric properties (e.g., 1 for right angles, 0 otherwise). The symbolic complexity is then the distance between a quadrilateral’s vector and that of its deviant. More details are provided in the original study (Sablé-Meyer et al., 2021).

### Data Analysis

We first used logistic regressions to compare error rates to chance levels for each grade. Next, to determine which model best explained children’s data, we performed two-parameters multiple linear regressions within each grade, where the predictors of both models were put in competition. Finally, we assessed the potential impact of the music program using both a frequentist binomial mixed-model regression and a Bayesian logistic regression. In both models, violin training (violin vs. control), grade, SPI, and the two models were included as predictors, with participants as a random effect.

### Results

#### Error Rates.

**Figure 4.**
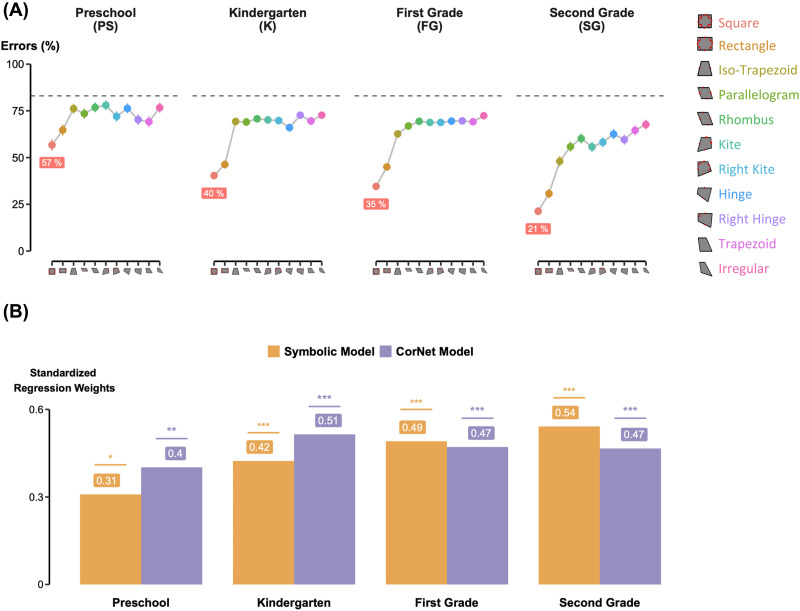
Geometric regularity effect in children. (A) Error Rates by Shape Regularity. Percentage of errors for each of the eleven quadrilaterals, averaged across subjects in each grade. Shapes are ordered by decreasing regularity as in Sablé-Meyer et al. (2021). (B) Model Comparison: Symbolic vs. simple CCNs. Standardized regression weights comparing the predictive power of a Symbolic Model (geometric primitives) against simple CORnet Model (simulating the ventral visual stream). While CORnet is a strong predictor in preschool and kindergarten, the Symbolic Model emerges as the superior account in 1st and 2nd Grade. Significance levels: •*p* < 0.1; *p < 0.05; **p < 0.01; ***p < 0.001.

Consistent with the previous two experiments, children’s performance improved across grades, with average error percentages decreasing from 70 ± 8% in PS, to 64 ± 7% in KG, 62 ± 7% in 1stG, and 51 ± 7% in 2ndG. All groups performed significantly below chance-level error rates (PS: *z* = 19, KG = 38.9, 1stG: 48.5, 2ndG: 49.9; all *p* < .001) (Figure 4A).

In line with Sablé-Meyer et al. (2021), the most regular quadrilaterals—*squares* and *rectangles*—stood out in children’s performance. For instance, among kindergarteners, error percentages for squares and rectangles remained below 47%, while exceeding 64% for other more irregular quadrilaterals. Despite this dichotomy, average performance was still well predicted by the order of geometrical regularity in all grades (linear regression on 11 points: *t*(9) = 1.93, *p* = .086 for PS; *t*(9) = 2.77, *p* = .022 for KG; *t*(9) = 3.16, *p* = .012 for 1stG; and *t*(9) = 3.84, *p* = .004 for 2ndG). However, when squares and rectangles were excluded from the analysis (linear regression on only 9 points), the regression was no longer significant (*t*(9) = −.41, *p* = .69 for PS; *t*(9) = .31, *p* = .77 for KG; *t*(9) = 1.4, *p* = .20 for 1stG; and *t*(9) = 2.03, *p* = .082 for 2ndG). This indicated that the linearity previously observed was mainly driven by the differences in performance between squares and rectangles versus the other quadrilaterals.

To better understand grade-related trajectories, we examined which of the two models introduced in Sablé-Meyer et al. (2021)—a simple CNN versus a symbolic model—best explained children’s performances. Specifically, we performed a two-parameters multiple regression in each grade, where the predictors of both models were put in competition (Figure 4B). Both significantly explained part of the children’s performance (*p* < .001 for both predictors, for children after preschool). Yet, while correlations with the CNN plateaued over time, those with the symbolic model rose significantly (standardized regression weights = .31, .42, .49 and .54 for the symbolic model respectively for PS, KG, 1stG, and 2ndG; and .40, .51, .47, .47 for the CNN). This suggests that children progressively abandon the shallow visual strategy in favor of an abstract strategy based on geometric rules.

#### Music Training.

As in Experiments 1 and 2, we performed both a frequentist binomial mixed-model regression and a Bayesian logistic regression. In both models, group (violins vs. controls), grade, models (Symbolic and CNN) and SPI were included as predictor variables, with participants as a random effect (Table 5). No main effect of group was observed (*p* = .63, BF_10_ = .15). The performance of violin children and controls were similarly explained by both the symbolic model (*p* = .44, BF_10_ = .047) and the CCN model (*p* = .99, BF_10_ = .034) (Supplementary Material Figure S3). These similarities remained stable across grades (*p* = .66, BF_10_ = .039 for Group × Grade × Symbolic; *p* = .26, BF_10_ = .065 for Group × Grade × CCN).

**Table 5.** Parameter estimates from a frequentist binomial mixed-effects regression and a Bayesian logistic regression: *Errors* ∼ *Group* * *Grade* * (*Symbolic* + *CNN* + *SPI*) + (1|*Subject*). The predictors Grade, Symbolic, CNN and SPI were standardized prior to the regression.

**Predictor**	**Estimate**	**Std. Error**	***Z*-value**	***P*-value**	**BF_10_**
(Intercept)	0.492	0.053	9.338	<.001	8.81e7
Group	−0.070	0.146	−0.478	.633	.15
**Grade**	**−0.347**	**0.045**	**−7.624**	<.001 (***)	**5.98e7**
**CCN**	**0.321**	**0.024**	**13.586**	<.001 (***)	**9.95e14**
**Symbolic**	**−0.321**	**0.024**	**−13.183**	<.001 (***)	**3.28e14**
**SPI**	**−0.138**	**0.048**	**−2.901**	.004 (**)	**2.66**
Group × Grade	−0.123	0.194	−0.634	.526	.21
Group × CCN	0.000	0.034	−0.005	.996	.034
Group × Symbolic	−0.027	0.035	−0.774	.439	.047
**Group × SPI**	**0.362**	**0.168**	**2.147**	.032 (*)	**1.44**
Grade × CCN	0.025	0.024	1.069	.285	.042
**Grade × Symbolic**	**−0.080**	**0.024**	**−3.272**	.001 (***)	**4.56**
**Grade × SPI**	**−0.144**	**0.062**	**−2.342**	.019 (*)	**.83**
Group × Grade × CCN	0.039	0.034	1.127	.260	.065
Group × Grade × Symbolic	−0.016	0.035	−0.442	.658	.039
Group × Grade × SPI	0.407	0.228	1.783	.075	.94

### Discussion

Sablé-Meyer et al. (2021) demonstrated that humans, unlike baboons, were sensitive to the regularity of geometric shapes, such as those defined by parallelism, right-angles or symmetries. In the same *odd-one-out* task, where participants had to identify an intruder among six quadrilaterals (one of which has a displaced vertex), adults performed more accurately when the reference shape was a regular quadrilateral (e.g., a square or a rectangle) than when it was a more irregular one, such as a trapezoid. In contrast, baboons showed comparable performance across all shapes, regardless of geometric regularity.

Our findings replicate and extend those of Sablé-Meyer et al. (2021), revealing a dual modulation shaped by both geometric regularity and grade, but not by musical practice. Children’s performance improved significantly with grade level and, critically, increased with the geometric regularity of the quadrilaterals. Although accuracy correlated significantly with the regularity rank of quadrilaterals (from square to completely irregular shapes), this effect was largely driven by the two most regular shapes: squares and rectangles. For example, kindergarteners made less than 47% errors with squares and rectangles, but between 64% and 75% with less regular shapes. When the two simplest shapes were excluded, the linear relationship between regularity and performance vanished, whereas it persisted in adults (Sablé-Meyer et al., 2021). Thus, the apparent linearity of the geometric effect may in fact reflect a stepwise learning process, potentially driven by familiarity: children might first grasp highly regular, frequently encountered shapes and only later extend this understanding to less regular ones. Further data from older children will be needed to determine whether this development is continuous or reflects discrete stages, possibly influenced by the acquisition of mathematical vocabulary.

To explain human and animal performance, Sablé-Meyer et al. (2021) contrasted two classes of models of geometric shape perception: simple convolutional neural networks such as CorNet (CNNs) that model early feedforward processing within the ventral visual pathway, and a symbolic model capturing the processing of nonaccidental geometric properties such as parallelism and angles. Recent neuro-imaging data demonstrated that, when adult participants viewed quadrilaterals, CNNs captured early visual activity in the occipital lobe, while the symbolic model accounted for subsequent dorsal parietal and prefrontal activations (Sablé-Meyer et al., 2026). These results confirm that simple CNNs model a shallow visual strategy localized to the visual cortex, whereas the symbolic model reflects an abstract, rule-based strategy involving higher-level areas. In our study, although both models accounted for a part of children’s performance, the variance explained by the symbolic model increased significantly with grade, while the explanatory power of simple CNNs remained essentially constant. This observation suggests that children progressively shifted between two complementary strategies to perform the intruder task. Initially, they relied mostly on a low-level visual strategy, treating geometric shapes like any other object, a strategy shared with nonhuman primates. As they progressed through grade levels, however, children increasingly adopted a more symbolic strategy, interpreting the shapes through a set of geometric properties—an approach beyond the reach of simple CNNs and nonhuman primates.

While a developmental trajectory was evident, it was not significantly influenced by musical practice. Overall performance was comparable between violin and control groups across all grades, and neither the symbolic nor the CNN model showed stronger correlations with either group. Thus, musically trained and untrained children appeared to rely on similar strategies. The abstract and recursive nature of both domains alone does not seem sufficient for musical practice to facilitate the acquisition of geometric abilities.

## EXPERIMENT 4: ATTENTION TASK

Experiment 4 evaluated the development of attentional selection and inhibitory control. Given the level of cognitive control required to play an instrument—particularly within an orchestra—we expected an effect of musical training, as reported in several previous studies (Bolduc et al., 2021; Bugos & DeMarie, 2017; Frischen et al., 2021; Guo et al., 2018; Holochwost et al., 2017; Janus et al., 2016; Moreno et al., 2011).

### Experimental Paradigm

In each trial, five animals were displayed in a row, and participants had to identify the direction the central animal was facing. If it faced left, they had to press the left button; if it faced right, they had to press the right button. The four surrounding distractor animals (see Figure 1C) influenced the task in different ways. On congruent trials, distractors faced the same direction as the central animal, while on incongruent trials, distractors faced the opposite direction, requiring participants to ignore them. On no-go trials, the distractors were different animals from the central one, signaling that participants should withhold their response. To increase task difficulty, no-go trials were deliberately made more than twice as infrequent as the other two trial types. This task, inspired by a previous study (Bunge et al., 2002), examines two types of attentional control (Posner & Petersen, 1990): *selective attention*, reflected by performance differences between incongruent and congruent trials, and *executive control*, reflected by performance differences between no-go and other trials. Each child completed 60 trials: 25 congruent, 25 incongruent and 10 no-go trials. Each trial lasted four seconds during which they could respond, with two seconds of stimulus presentation followed by two seconds of a gray background during which participants could respond.

### Data Analyses

We first conducted one-sample *t*-tests to compare each grade’s error rates to chance level. To evaluate differences in performance and response times between congruent and incongruent trials, we ran, within each grade, a mixed-model regression with condition (congruent vs. incongruent) as the predictor and participants included as a random effect. To assess the potential impact of the music program, we applied both a frequentist binomial mixed-model regression and a Bayesian logistic regression, on performance and reaction times. In both models, violin training (violin vs. control), condition (congruent vs. incongruent), grade, and SPI were included as predictors, with participants as a random effect. For incongruent and congruent trials, pressing the wrong button or failing to respond was treated as an error, as in Bunge et al. (2002). To evaluate performance on no-go trials, we computed d-prime scores, defined as the difference between the *z*-transformed *hit rate* (responses on go-trials: congruent and incongruent) and the *z*-transformed *false alarm* (responses on no-go trials). Higher d-prime values indicate greater sensitivity. We performed a linear regression on d-prime scores, with groups (violins vs. control), SPI, and grade as predictor, to assess the impact of the music program on executive control abilities.

### Results

#### Error Rates.

**Figure 5.**
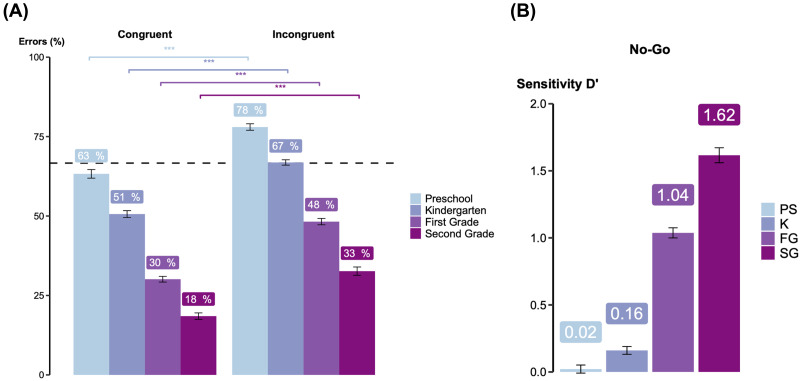
Development of attentional abilities across school grades. (A) Percentage of errors obtained in congruent and incongruent trials, in each grade. Stars indicated significance level from mixed-model binomial regressions performed in each grade: *Errors * ∼ *Condition* + (1|*Subject*). (B) D-prime scores, computed as *z*(*hit rate*) − *z*(*false alarm*), within each grade.

Performance gradually improved with grade level on both congruent and incongruent trials. Specifically, error percentages on congruent trials decreased from 63 ± 13% to 51 ± 13%, 30 ± 12% and 18 ± 10% and on incongruent trials from 78 ± 9%, 67 ± 10%, 48 ± 13% and 33 ± 13%, respectively for PS, KG, 1stG and 2ndG (Figure 5A). Notably, only the youngest children (PS) failed to perform above chance level on incongruent trials (congruent trials: *t*(83) = 2.48, *p* = .008 for PS, *t*(153) = 8.93, *p* < .001 for K, *t*(178) = 22.29, *p* < .001 for 1stG, *t*(97) = 25.06, *p* < .001 for 2ndG; incongruent trials: *t*(83) = −3.92, *p* = 1 for PS, *t*(153) = 1.85, *p* = .033 for K, *t*(178) = 10.95, *p* < .001, *t*(97) = 14.18, *p* < .001). Across all grades, children performed significantly better on congruent trials than on incongruent ones (*p* < .001 for each grade).

#### Response Times.

RTs remained globally stable across grades in congruent trials, averaging 1,305 ms for PS, 1,316 ms for KG, 1,319 ms for 1stG, and 1,298 ms for 2ndG. In contrast, RTs increased with grade in incongruent trials, averaging 1,348 ms for PS, 1381 ms for KG, 1,407 ms for 1stG, and 1,433 ms for 2ndG (Supplementary Material Figure S4C). Children from preschool onward responded significantly faster on congruent than on incongruent trials (*p* = .14 for PS; *p* < .001 for all other grades).

#### Music Training.

As in the previous experiment, we conducted both a frequentist binomial mixed-model regression and a Bayesian logistic regression. In both models, group (violins vs. controls), grade, condition (congruent vs. incongruent), and SPI were included as predictors, with participants as a random effect (Table 6). Overall, control children made, surprisingly, fewer errors than violin ones (*p* = .002, BF_10_ = 7.65), although this difference decreased with grade (*p* = .011, BF_10_ = 2.61) (Supplementary Material Figure S4A). However, control children were more affected by incongruency than violin students: children learning the violin made fewer errors than controls on incongruent trials compared to congruent trials (*p* = .037). The Bayesian analysis, however, indicated only anecdotal evidence for this difference (BF_10_ = .49 < 1, i.e., favoring the null hypothesis) (Supplementary Material Figure S4A). Interestingly, violin children were less impacted than control ones by SPI (*p* = .008, BF_10_ = 2.62). Finally, the three-way interaction between group, grade, and condition was not significant (*p* = .13, BF_10_ = .21), indicating no additional grade-related differentiation of congruent and incongruent trials in favor of the violin group.

**Table 6.** Coefficients obtained from both a frequentist binomial mixed model regression and a Bayesian logistic regression, performed on congruent and incongruent trials: *Errors* ∼ *Group* * *Grade* * (*Condition* + *SPI*) + (1|*Subject*). The Grade and SPI predictors were standardized prior to the regression.

**Predictor**	**Estimate**	**Std. Error**	***Z*-value**	***P*-value**	**BF_10_**
(Intercept)	−0.515	0.102	−5.041	<.001	130.20
**Group**	**−0.907**	**0.293**	**−3.095**	.002 (**)	**7.65**
**Grade**	**−1.008**	**0.089**	**−11.316**	<.001 (***)	**3.26e12**
**Condition**	**0.856**	**0.043**	**20.131**	<.001 (***)	**2.63e25**
SPI	−0.059	0.090	−0.656	.512	.11
**Group × Grade**	**−1.100**	**0.430**	**−2.557**	.011 (*)	**2.61**
**Group × Condition**	**0.128**	**0.061**	**2.085**	.037 (*)	**.49**
**Group × SPI**	**0.897**	**0.337**	**2.665**	.008 (**)	**2.62**
**Grade × Condition**	**0.117**	**0.044**	**2.642**	.008 (**)	**1.55**
Grade × SPI	0.086	0.118	0.733	.463	.16
Group × Grade × Condition	−0.096	0.064	−1.506	.132	.21
**Group × Grade × SPI**	**1.477**	**0.503**	**2.938**	.003 (**)	**10.57**

The results of a similar mixed-model linear regression performed on response times (only correct responses) are presented in Table 7, with no effect related to music training (Supplementary Material Figure S4D).

**Table 7.** Coefficients obtained from both a frequentist linear mixed model regression and a Bayesian linear regression performed on congruent and incongruent trials: *RTs* ∼ *Group* * *Grade* * (*Condition* + *SPI*) + (1|*Subject*). The Grade and SPI predictors were standardized prior to the regression.

**Predictor**	**Estimate**	**Std. Error**	***t*-value**	***P*-value**	**BF_10_**
(Intercept)	1310.713	15.233	86.042	<.001 (***)	3.21e192
Group	−83.028	53.606	−1.549	.122	.99
Grade	−8.748	13.305	−0.657	.511	1.05
**Condition**	**84.127**	**8.030**	**10.477**	<.001 (***)	**340.42**
SPI	−3.811	13.457	−0.283	.777	.99
Group × Grade	−121.156	84.866	−1.428	.154	1.00
Group × Condition	17.613	11.219	1.570	.116	4.47
Group × SPI	102.356	62.874	1.628	.104	.99
**Grade × Condition**	**34.061**	**7.889**	**4.317**	<.001 (***)	**9.36**
Grade × SPI	−3.356	17.721	−0.189	.850	1.04
Group × Grade × Condition	−2.613	11.390	−0.229	.819	1.44
Group × Grade × SPI	142.066	99.062	1.434	.152	1.05

#### Executive Control (No-Go Trials).

On average, preschoolers responded on 47% of go trials (congruent + incongruent) and 44% of no-go trials; kindergarteners on 58% of go trials and 51% of no-go trials; first graders on 73% of go trials and 39% of no-go trials; and second graders on 81% of go trials, and 29% of no-go trials. To evaluate these performances, we computed d-prime scores, defined as the difference between the *z*-transformed *hit rate* (responses on go trials) and the *z*-transformed *false alarm* (responses on no-go trials). As with other measures, children’s sensitivity on no-go trials improved with grade, with d-prime scores (averaged across participants) of 0.02 in PS, 0.16 in KG, 1.04 in 1stG, and 1.62 in 2ndG (see Figure 5B). Only preschoolers’ d-prime scores were not significantly above zero (PS: *t*(83) = 0.36, *p* = .72; KG: *t*(153) = 2.77, *p* = .006; 1stG: *t*(178) = 13.68, *p* < .001; 2ndG: *t*(97) = 14.41, *p* < .001; one-sample *t*-tests).

A linear regression performed on d-prime scores, with group (violins vs. control), grade, and SPI as the predictor, revealed no significant difference between violin and control children (Table 8 and Supplementary Material Figure S4B). Running same regression separately on d-prime scores computed from congruent-only or incongruent-only trials produced the same pattern of results.

**Table 8.** Coefficients obtained from a regression performed on d-prime scores: *D*′ ∼ *Group* * *Grade* * *SPI*. The grade and SPI predictors were standardized prior to the regression.

**Predictor**	**Estimate**	**Std. Error**	***t*-value**	***P*-value**	**BF_10_**
(Intercept)	0.634	0.071	8.904	<.001	5.98e8
Group	0.089	0.179	0.498	.619	.20
Grade	0.505	0.061	8.273	<.001 (***)	9.03e6
SPI	−0.049	0.064	−0.766	.444	.086
Group × Grade	0.194	0.246	0.788	.431	.30
Group × SPI	0.114	0.204	0.557	.578	.23
Grade × SPI	−0.167	0.083	−2.005	.046 (*)	.60
Group × Grade × SPI	0.034	0.289	0.117	.907	.26

### Discussion

We found significant developmental grade-level improvements in both selective and executive attention abilities. The youngest children (preschoolers) made over 75% errors on incongruent trials, while second graders made less than 35%, illustrating a substantial improvement in their ability to inhibit irrelevant peripheral stimuli and focus on the central target. As expected, children across all grades performed worse on incongruent trials than on congruent trials, making significantly more errors and responding more slowly. Similarly, children became more sensitive, responding more frequently when a response was expected (go trials) and withholding more and more responses when none was expected (no-go trials). The developmental trajectory of these attentional abilities aligns with prior research (Boen et al., 2021; Rueda et al., 2004).

When comparing musically trained and untrained children, no robust advantage of violin practice emerged. Although violin children tended to commit slightly fewer errors than controls on incongruent versus congruent trials, Bayesian analyses indicate that this effect was weak and, in fact, largely explained by an unexpected drop in their accuracy on congruent trials. Indeed, in this condition, violin children made significantly more errors than controls. Thus, if musical training exerts any influence on visual selective attention, as the interaction might suggest, this effect appears limited and warrants further investigation. With respect to executive control, the two groups did not differ. This null effect is unlikely due to a lack of sensitivity, as strong grade-related differences were readily detected, suggesting that musical training did not influence executive control abilities in our sample.

This result may appear surprising, as executive control is central to instrumental practice: playing an instrument is a demanding motor activity that requires constant inhibition of automatic or unwanted movements. Nevertheless, our non-significant results are compatible with the existing literature, in which evidence for such an effect remains mixed. Among studies using the Go/No-Go paradigm, only one reported a clear positive influence of musical learning on executive control (Holochwost et al., 2017), and it involved an intensive program with two hours of daily training. Other studies have reported mixed (Bolduc et al., 2021; Hallberg et al., 2017; Moreno et al., 2011) or null results (Guo et al., 2018). Similarly, in tasks tapping verbal inhibition, such as the Day/Night Stroop, only the study without an active control group found positive effects (Bugos & DeMarie, 2017; Frischen et al., 2021; Shen et al., 2019).

Selective visual attention has been less frequently examined in this context. One study, using a flanker task, reported positive effects of music training (Holochwost et al., 2017), whereas two other studies employing other selective visual attention tasks found negative results (James et al., 2020; Janus et al., 2016). Future work examining a broader range of attentional processes, including the relatively underexplored domain of auditory selective attention, which may be more directly shaped by musical practice, could provide deeper insight into the specific cognitive abilities most likely to benefit from musical training.

## CORRELATIONS BETWEEN EXPERIMENTS

We next examined whether performance across tasks was interrelated—specifically, whether a child who performed well in one task (e.g., attention) also performed well in another (e.g., geometric reasoning). Pearson correlations between children’s average accuracies revealed that inhibition performance significantly predicted performance in both visual tasks (*r* = 0.46, *p* < 0.001 with visual abstraction; *r* = 0.35, *p* < 0.001 with quadrilaterals). The two visual tasks were also moderately correlated (*r* = 0.33, *p* < 0.001), whereas correlations involving the auditory sequence task were weaker (*r* = 0.15–0.29, all *p* < 0.001). Across grades, the link between attention and visual abstraction strengthened steadily (*r* = 0.09 to 0.42 from preschool to second grade), suggesting a developmental coupling between attentional control and visual chunking abilities.

To determine whether children who used symbolic strategies in one task tended to do so in others, we correlated the standardized regression weights associated with the symbolic models across tasks (LoT-Chunk in auditory and visual tasks, and the symbolic model in geometrical task). Overall, cross-task correlations were weak or negligible: notably, LoT-Chunk weights in the auditory and visual tasks were uncorrelated, indicating distinct strategies across modalities as indicated by the group analyses. More interestingly, a modest link emerged between symbolic reasoning in the geometrical task and LoT-based encoding in the auditory task. Children whose performance on the quadrilateral task was best captured by the symbolic model (R^2^ > 1)—as opposed to the CorNet model—were also those for whom the LoT-Chunk model was a significantly better predictor than the Chunk model in the auditory task (*t*(118) = 3.61, *p* < .001, from linear regression) (Supplementary Material Figure S5). In contrast, children whose geometrical performance was better explained by the CorNet model showed no such difference between Lot-Chunk and Chunk models in the auditory task (*t*(86) = 1.90, *p* = 0.24). No significant differences were found for either group in the visual abstraction task (CorNet children: *t*(86) = −.010, *p* = .92, Symbolic children: *t*(124) = −.71, *p* = .48).

To summarize, cross-task analyses suggest that children do not rely on a single, domain-general symbolic strategy but instead develop symbolic representations at different rates across modalities. Interestingly, symbolic reasoning in geometry appeared to co-occur with the use of structured, LoT-like representations in auditory sequence learning, whereas visual abstraction remained largely non-symbolic. This pattern supports the idea that the acquisition of symbolic reasoning emerges progressively and unevenly across cognitive domains, with auditory and geometric structures providing the earliest grounds for a shared “language-of-thought” system.

## GENERAL DISCUSSION

### Development of Symbolic Reasoning in Early Childhood

Children from preschool to second grade completed three experiments designed to explore the development of their abstraction abilities across different domains. By first grade, children began encoding complex auditory sequences using a Language of Thought (LoT), forming hierarchical and structured representations similar to those observed in adults. In contrast, visual patterns were processed differently with children relying more on chunking strategies rather than symbolic compression. This may reflect a later emergence of abstract visual processing, a modality-specific effect or a task-dependent limitation, with children focusing only on the last four items of the sequence—where the changes occur—to perform the task. Finally, when confronted with quadrilaterals, children showed increasing sensitivity to geometric regularities, indicating a developmental transition from perceptual to symbolic reasoning.

Across all experiments, particularly Experiments 1 and 3, children gradually moved from basic perceptual grouping to more structured, rule-based thinking, reflecting the progressive development of abstract cognition. However, the present data do not allow us to isolate the respective contributions of neural maturation from those of pedagogical exposure. Formal schooling may facilitate this shift by providing both a conceptual framework and a specialized vocabulary that directs attention toward the abstract properties required for symbolic encoding, even when encountering novel tasks outside the traditional curriculum. These findings contribute to a growing body of research highlighting children’s early ability to encode both linguistic and non-linguistic information in abstract ways (Alderete et al., 2024; Cesana-Arlotti et al., 2018; Ciccione et al., 2023, 2026; Dautriche & Chemla, 2025; de Carvalho & Dautriche, 2025; Feiman et al., 2022; Mills et al., 2024; Piantadosi et al., 2012; Pomiechowska et al., 2024; Sablé-Meyer et al., 2021). Importantly, cross-task analyses indicated that symbolic reasoning in geometry co-occured with the use of structured, LoT-like representations in auditory sequence learning, pointing to the early emergence of a shared « language-of-thought » system in human reasoning (Dehaene et al., 2022).

### Impact of Musical Practice

We also investigated the impact of musical practice, as implemented in the program *A Violin in my school*, on the development of attention and abstraction abilities. These four experiments were designed to probe abilities that are theoretically increasingly distant from those explicitly involved in music training: from attentional control to auditory and visual abstraction, and finally to geometric reasoning.

Our results suggest that musical practice, as implemented in this program, did not significantly affect children’s abstraction abilities—whether auditory, visual, or geometric. In other words, the abstract, symbolic, and recursive nature of music does not seem to be sufficient to facilitate the acquisition of general-purpose abstraction abilities. This conclusion echoes prior research showing limited or no transfer of musical learning to mathematics or general intelligence (Bilhartz et al., 1999; Costa-Giomi, 2004; Schellenberg, 2004). For instance, Bilhartz et al. (1999), found that 4- and 5-year-olds who were randomly assigned to musical instruction outperformed control groups on visual a short-term memory test, but not on formal mathematical assessments. Similarly, Costa-Giomi (2004) who followed 117 children over three years, found no improvement in overall academic achievement, including math, among those taking piano lessons. More recent randomized studies have confirmed these observations, showing little to no effect of musical practice on math abilities compared to other artistic activities (Mehr et al., 2013; Rickard et al., 2012). More nuanced results exist however (Holmes & Hallam, 2017; Holochwost et al., 2017). For instance, Holochwost et al. (2017) observed that children who participated in an intensive music program (2 hours a day) achieved higher math and language grades, especially when enrolled in the program for multiple years. Yet, these effects may reflect the program’s intensity rather than music training per se. Furthermore, the absence of an active control group in this study prevents ruling out similar benefits from other demanding activities.

A similar pattern emerges for general intelligence. Numerous studies comparing music with other activities—such as dance, painting, reading or second-language lessons learning—report no significant effect of music training on general intelligence (D’Souza & Wiseheart, 2018; Flaugnacco et al., 2015; Janus et al., 2016; Nan et al., 2018). Studies suggesting small gains often present methodological limitations (Barbaroux et al., 2019; James et al., 2020), have been challenged (Schellenberg, 2004; Steele, 2005), or lack adequate control groups (Barbaroux et al., 2019; Osborne et al., 2016). Overall, the present findings add to the growing evidence that far transfer from musical to non-musical domains remains elusive and may depend on specific contextual or instructional factors. By demonstrating an absence of transfer to fundamental abstraction abilities—rather than merely to academic or standardized indicators as has largely been the case to date—our results offer a more stringent challenge to the hypothesis that musical practice facilitates the acquisition of general-purpose abstraction abilities.

Several factors may explain the absence of a measurable effect of musical training on abstraction abilities in our study. First, the *A Violin in My School* program, which targets children aged 4 to 8, primarily focuses, particularly with the younger kids, on the practical aspects of musical performance—learning rhythm, coordination, and ensemble playing—while only briefly introducing symbolic notation. Manipulation of symbolic components, such as the one involved in reading music or understanding musical structure, is introduced formally only two years in the program, with more advanced instruction involving solfeggio and music theory. Consequently, the type of symbolic manipulation that could support transfer to abstract reasoning may not yet be engaged in this early phase of learning. Second, the duration and developmental timing of the intervention may also have limited its effects. Four years of weekly group lessons provide valuable exposure to music but may not suffice to produce measurable changes in symbolic reasoning at this young age. Older children, or those receiving more intensive and individualized training, might show different outcomes. Yet, the few longitudinal or randomized studies available in such populations have reported mixed or inconclusive results (Holochwost et al., 2017; Rickard et al., 2012).

While our findings challenge the existence of far transfer between music and abstract reasoning, they do not exclude more proximal effects. In Experiment 1, we observed a slight, though non-significant, advantage of musical training on auditory working memory, and in Experiment 4, a modest enhancement in visual selectivity. However, no measurable effect was found on executive control. These results align with the broader literature, in which evidence for an impact of musical training on executive functions remains mixed (Bolduc et al., 2021; Bugos & DeMarie, 2017; Frischen et al., 2019, 2021; Guo et al., 2018; Hallberg et al., 2017; Holochwost et al., 2017; James et al., 2020; Janus et al., 2016; Linnavalli et al., 2018; Moreno et al., 2011; Schellenberg & Lima, 2024; Shen et al., 2019). The fact that these studies vary widely methodologically, often involving small sample sizes and a limited range of tasks, makes the generalizations of their results highly challenging and uncertain. However, recent reviews conclude that randomized studies with active control groups generally fail to find evidence for an improvement in executive functions following musical training (Schellenberg & Lima, 2024), though some meta-analyses suggest modest positive effects (Jamey et al., 2024).

The most compelling evidence for cross-domain transfer comes from studies explicitly linking mathematics and music, by leveraging the former to teach specific mathematical concepts. For example, explicit teaching of rhythmic structure has been shown to improve children’s understanding of mathematical fractions (Azaryahu et al., 2020; Courey et al., 2012; Ribeiro & Santos, 2017). These reports suggest that transfer arises not automatically from musical engagement but when educators explicitly highlight shared principles between domains (Guilmois et al., 2019; Mayer, 2004; Stockard et al., 2018). Thus, beyond the intrinsic pleasure of learning to play music, any academic benefit is likely to arise only when musical concepts are explicitly linked to analogous mathematical ones—for instance, highlighting the connections between musical notes, fractions, and powers of two. In this sense, our results reinforce broader evidence that unguided discovery learning rarely promotes the generalization of abstract rules in young children (Guilmois et al., 2019; Mayer, 2004; Stockard et al., 2018).

Finally, it is important to note that we only tested non-musical abilities in this study. However, as previously proposed (Mehr, 2025; Peretz & Coltheart, 2003), musical training could primarily and above all refine fundamental skills specific to music—such as the processing of tonal and metrical hierarchies—rather than general sequential or geometric reasoning. Future research utilizing tasks sensitive to these music-specific structural congruencies may reveal strong gains within the musical domain, even in the absence of the ‘far transfer’ effects investigated here.

### Limitations of the Program

The *A Violin in My School* program had already been implemented before the present study was planned. Our research therefore took the form of a natural experiment, making use of the unique opportunity provided by this large-scale educational initiative deployed in schools located in socioeconomically disadvantaged areas. This real-world setting offered exceptional ecological validity but came with several methodological limitations.

A first limitation concerns the randomization procedure. Randomization was not performed at the school level, leaving open the possibility that pre-existing differences between schools acted as confounding factors. We attempted to control for this bias by including each school’s social position index as a covariate in our regressions. Yet, this index provides only a limited proxy for socioeconomic diversity. Ideally, randomization should have been implemented at the school or even at the classroom level, but the practical circumstances surrounding the deployment of the program did not permit it.

A second limitation concerns the nature of the intervention itself. Violin lessons were held during school hours, replacing time that would have otherwise been devoted to other school subjects. In principle, control children therefore received more traditional instruction. However, because each teacher was free to organize their schedule, the affected academic domains likely varied across classrooms and schools. To identify which areas were most affected, a sociological survey conducted in 2022–2023 by Pereira (2025) gathered responses from 123 of the 153 first-grade teachers involved in the program. Over half on the violin instruction classroom teachers reported spending less time on visual arts (56%), world discovery (51%), or foreign languages (45%), while fewer teachers reported spending less time on mathematics (16%) and French (12%). These reports suggest that mathematics, the domain most directly relevant to our experiments, was relatively preserved. However, this conclusion should be interpreted with caution given that the survey covered only one age group and relied on self-reported estimates. In sum, the absence of differences between violin students and control children could partly reflect differences in instructional time rather than a genuine lack of transfer from musical practice to the measured abilities. While this concern cannot be excluded, the survey data provide some reassurance that mathematics was not disproportionately affected. Future studies would benefit from including clearly defined active control conditions, as done in previous works (e.g., D’Souza & Wiseheart, 2018; Frischen et al., 2019, 2021)

Finally, the abilities assessed in our study—mainly abstract reasoning and pattern learning—cannot easily be classified as “near” or “far” relative to either schooling or musical training. For instance, in the auditory abstraction task, children were asked to memorize and compare short melodies, an exercise directly aligned with the objectives of the violin program (e.g., “memorize a simple melody or short rhythmic pattern”). In this sense, our tasks may be at least as close to musical training as to standard academic instruction. While the degree of proximity between tasks and training surely varies across our paradigms, framing the results as a comparison between near transfer in control children and far transfer in violinists would thus be misleading.

### Conclusion

Taken together, our results highlight the gradual emergence of abstract representations during the first years of schooling, supporting the view that symbolic reasoning is shaped more by educational experience than by early exposure to structured activities such as music. The study also underscores the value—and the challenges—of investigating cognitive development in real-world educational contexts. Future work should aim to identify which forms of instruction most effectively promote the transition from perceptual or procedural learning to genuine abstract reasoning, and how explicit connections between domains such as music, language, and mathematics might foster this process.

## ACKNOWLEDGMENTS

We sincerely thank Fosca Al Roumi, Samuel Planton and Mathias Sablé-Meyer for their valuable input in adapting their paradigms and analyses. We also thank Claire Njoo Deplante for her useful suggestions on the manuscript. We are also grateful to the children who participated.

## CONFLICT OF INTERESTS

This research was supported by the Vareille Foundation, which provided funding for the experimental work and the doctoral fellowship of Théo Morfoisse. The foundation is the developer and funder of the educational program *Un Violon dans mon école*; however, it had no role in the scientific study design, data collection, analysis, interpretation of the results, or the decision to submit the work for publication. Conversely, the authors were not involved in the design or implementation of the *Un Violon dans mon école* program.

## FUNDING INFORMATION

This research was generously funded by the Vareille Foundation. Additionally, our broader research program on child development is supported by The Robert Debré Child Brain Institute which receives funding from the French government managed by the Agence Nationale de la Recherche (ANR) as part of the France 2030 program (reference ANR-23-IAHU-0010), and by an ERC grant MathBrain (ERC-2022-ADG101095866) to S.D.

## AUTHOR CONTRIBUTIONS

T.M.: Conceptualization; Data curation; Formal analysis; Writing – original draft G.D.L.: Conceptualization; Funding acquisition; Supervision; Writing – review & editing S.D.: Conceptualization; Formal analysis; Writing – original draft; Writing – review & editing S.B.: Writing – review & editing; M.P.: Writing – review & editing; C.P.W.: Writing – review & editing.

## DATA AVAILABILITY STATEMENT

All pre-recorded instructional videos and scripts for the analyses are available on the OSF platform: https://osf.io/h8yk7/overview?view_only=c0f08a0ef95f489984c43d2ed4ba34b2.

## Supplementary Material


