# Supplementary material for: Impact of Education and Music Training on the Development of Abstract Thinking in the First Years of Schooling
Source: Open Mind (Camb). 2026 May 29;10:707–38. doi: 10.1162/OPMI.a.354 (PMC13233093; doi:10.1162/OPMI.a.354)
Supplement: Supplementary file 1 [file opmi-10-707-s001.pdf]

Supporting information for

Impact of education and music training on the development of abstract thinking in the first years of schooling

Morfoisse Théo[1,2], Séverine Becuwe[1], Marie Palu[1], Cassandra Potier Watkins[2], Ghislaine Dehaene-Lambertz[1], Stanislas Dehaene[1,2]

[1]Collège de France, 11 Place Marcelin Berthelot, 75005 Paris, France
[2]Cognitive NeuroImaging Unit, CEA, Inserm, Université Paris-Sud, Université Paris-Saclay, NeuroSpin Center, 91191 Gif/Yvette, France

Corresponding author: Théo Morfoisse
Email: theo.morfoisse@gmail.com

This PDF file includes:

Figures S1 to S6
Table S1

Supplementary Figures

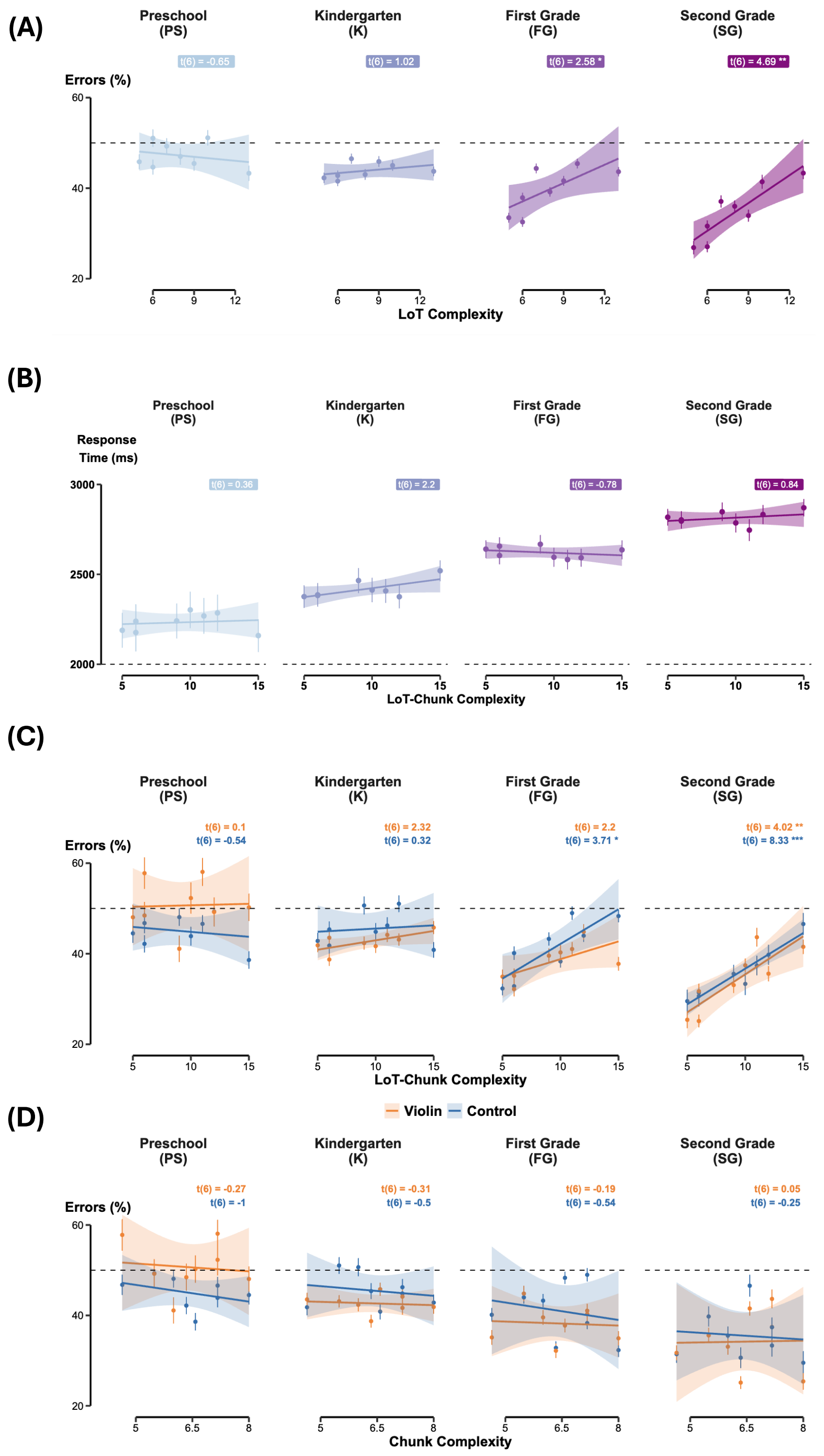

Supplementary Figure 1. Auditory sequences. (A) Percentage of errors in each sequence, averaged across all subjects within each grade, as a function of LoT complexity. (B) Response times in each sequence, averaged across all subjects within each grade, as a function of LoT-Chunk complexity. (C-D) Percentage of errors in each sequence, averaged across all subjects within each grade, separately for violin and control children, as a function of LoT-Chunk complexity (C), and Chunk complexity (D).

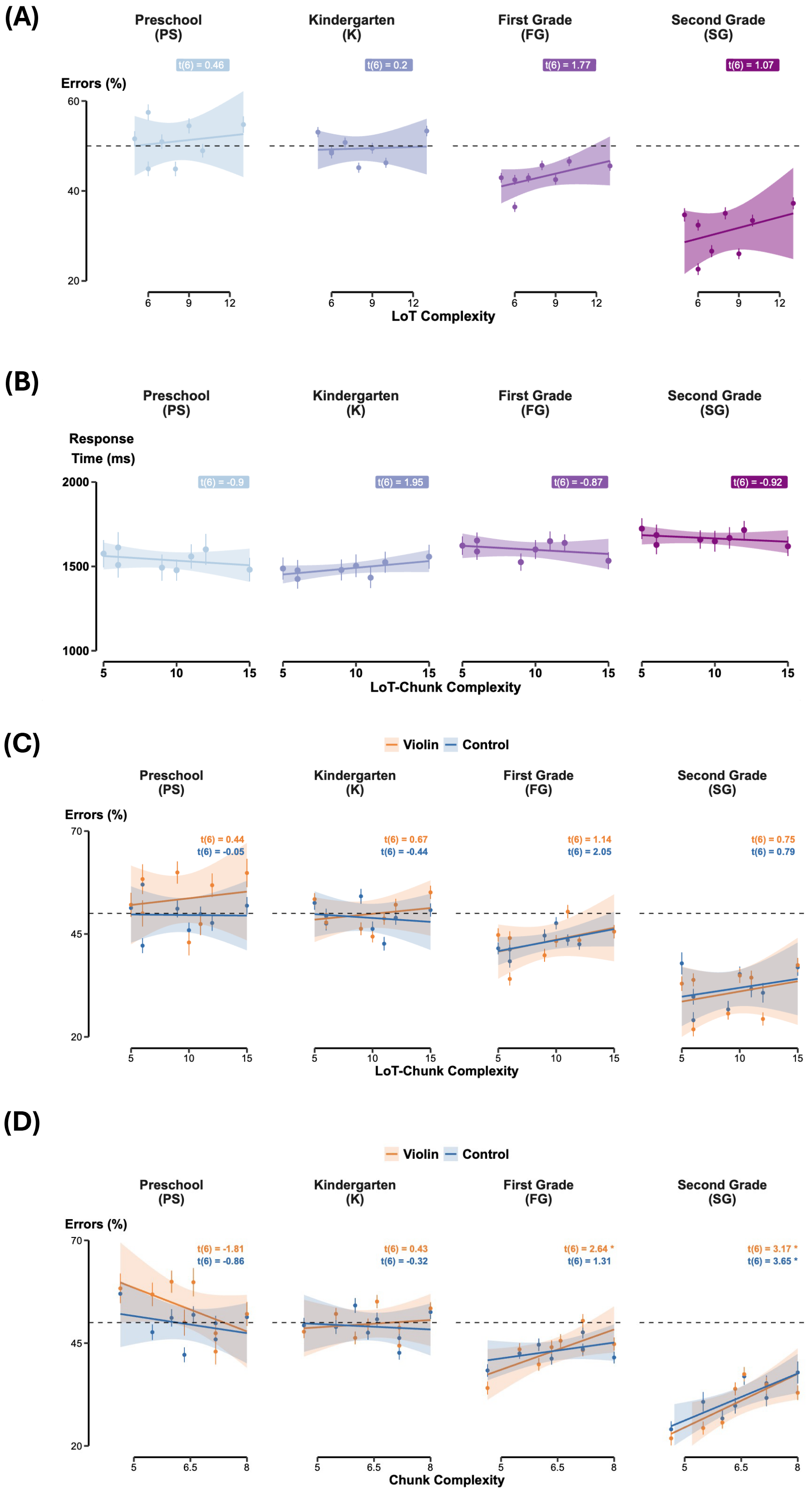

Supplementary Figure 2. Visual patterns (A) Percentage of errors in each pattern, averaged across all subjects within each grade, as a function of LoT complexity. (B) Response times in each pattern, averaged across all subjects within each grade, as a function of LoT-Chunk complexity. (C-D) Percentage of errors in each pattern, averaged across all subjects within each grade, separately for violin and control children, as a function of LoT-Chunk complexity (C), and Chunk complexity (D).

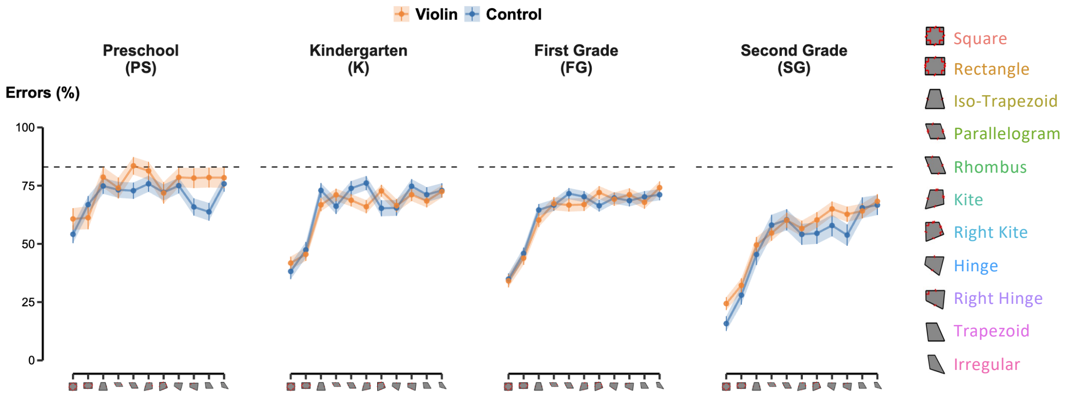

Supplementary Figure 3, Quadrilaterals. Percentage of errors for each quadrilateral, averaged across all subjects in each grade, separately for violin and control children. Shapes are ordered as in Sablé-Meyer et al. (2021).

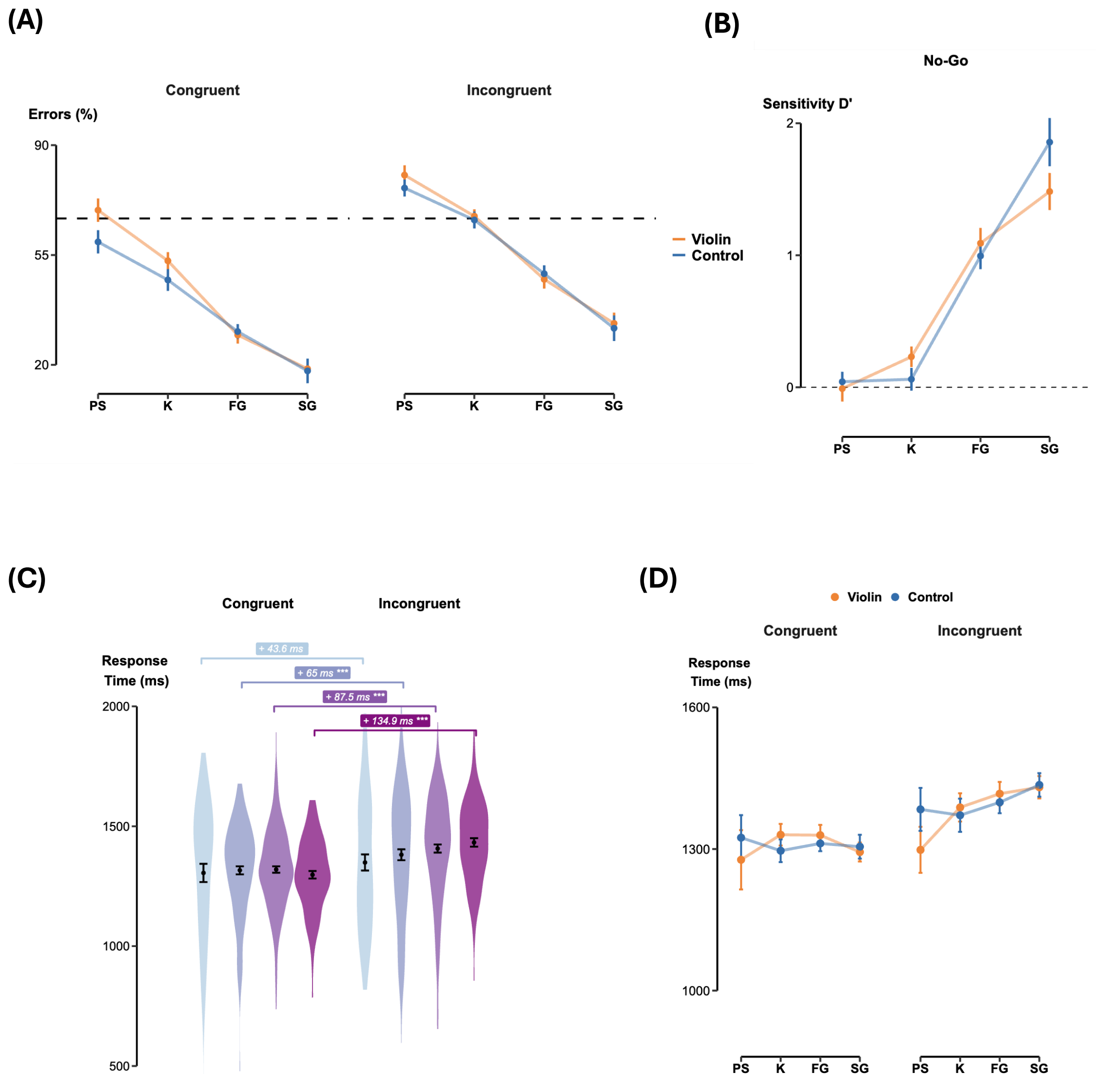

Supplementary figure 4. Attention. (A) Percentage of errors obtained in congruent and incongruent trials, in each grade, separately for violin and control children. (B) D-prime scores, computed as $z(hit\ rate) - z(false\ alarm)$, within each grade, separately for violin and control children. (C) Response Times in congruent and incongruent trials, in each grade. Stars indicated significance level from mixed-model binomial regressions performed in each grade: $RTs \sim Condition + (1|Subject)$. (D) Same as (C) but shown separately for violin and control groups.

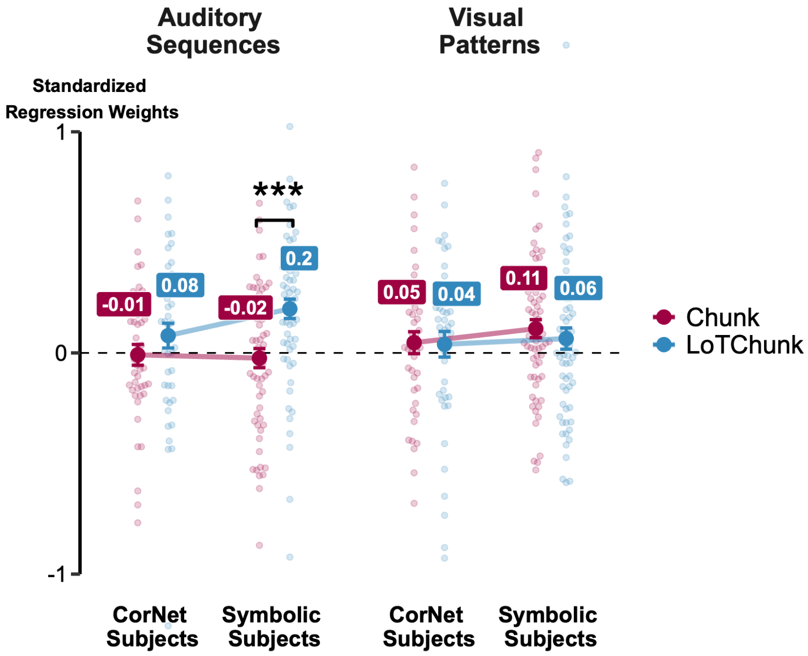

Supplementary figure 5. Symbolic strategies common to quadrilateral and auditory sequences tasks. Subjects were separated into three groups: those whose performance in the quadrilateral task was largely predicted by the symbolic model, those by the cornet model, and others. For the first two groups, the regression weights between their performance and predictions of LoT-Chunk and Chunk models in auditory sequences and visual patterns were plotted. Stars indicate whether the regression weights between the two groups of children were significantly different (•P < 0.1; *P < 0.05; **P < 0.01; ***P < 0.001).

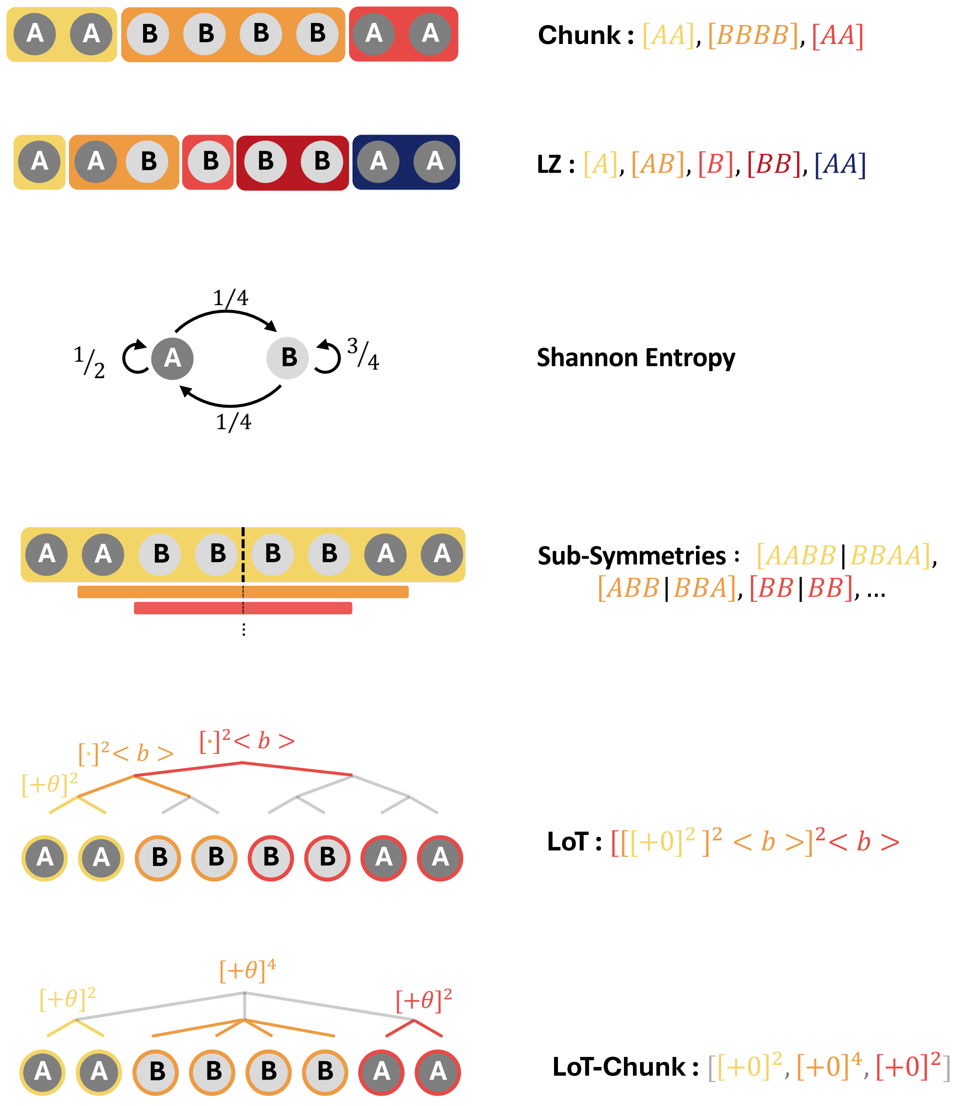

Supplementary Figure 6. Illustrations of the competing models for pattern encoding.

Supplementary Table

	School	SPI	Number of children tested
Preschool & Kindergarten	Violon 1	85.7	39
	Violon 2	88.7	52
	Violon 3	75	38
	Control 1	90.9	42
	Control 2	75.9	32
	Control 3	90.6	47
1st and 2nd Grades	Violon 1	82.6	46
	Violon 2	86.9	39
	Violon 3	73.6	87
	Control 1	91.5	28
	Control 2	91.3	59
	Control 3	90.3	52

Supplementary table 1. Number of children tested per school and each school’s social position index (SPI).
